# Direct evidence for cell adhesion-mediated radioresistance (CAM-RR) on the level of individual integrin β1 clusters

**DOI:** 10.1038/s41598-017-03414-4

**Published:** 2017-06-13

**Authors:** Laura Babel, Miriam Grunewald, Robert Lehn, Markus Langhans, Tobias Meckel

**Affiliations:** 1 Membrane Dynamics, Department of Biology, Technische Universität Darmstadt, Darmstadt, Germany; 2 GRK 1657, Molecular and cellular responses to ionizing radiation, Technische Universität Darmstadt, Darmstadt, Germany

## Abstract

The cellular interaction with the extracellular matrix (ECM) modulates many key processes such as proliferation, migration, differentiation and survival. In addition, cells cultured under 3D conditions in presence of an ECM display a marked radioresistance towards ionizing radiation (IR) in comparison to conventionally 2D cultured cells. This process, also known as “cell-adhesion-mediated-radio-resistance” (CAM-RR), has been linked to the chromatin structure that differs between cells cultured on stiff surfaces versus cell grown on soft planar supports or in 3D environments. As integrins are the key mediators of cell adhesion and mechanosensing, they originate the molecular signalling towards chromatin remodelling in response to a cell’s microenvironment. We aimed to investigate this molecular origin that leads to CAM-RR by investigating the distribution of integrins at the single molecule level and show that cells cultured in 2D keep a lower fraction of integrin β1 in clusters and maintain a less defined cluster status than 3D cultured cells. Upon X-irradiation this nanoscale distribution of integrin β1 is disturbed at much lower dosages in 2D versus 3D cultured cells. Radioresistance is thus linked to the ability to maintain a well defined organization of integrins in clusters, making integrin distribution a potential drug target for radiosensitization.

## Introduction

It is now well accepted that the microenvironment of cells has a profound impact on their physiology, which traditional two dimensional cell cultures are unable to provide^1–7^. In particular, cells cultured on a flat and rigid support lack three important aspects, which are key parameters for the physiological communication of cells with their environment^8, 9^. First, they lack dimensionality in that they do not allow cells to adhere to extracellular supports or adjacent cells with their entire surface, second, they provide a highly polarized rather than homogeneous mechanical environment and third, they lack the ability to maintain local concentration heterogeneities, e.g. gradients of soluble compounds. All mentioned parameters, namely (i) the distribution and density of adhesion sites on the extracellular matrix (ECM) or receptors on neighbouring cells, (ii) their mechanical resilience and (iii) local concentrations of solutes are processed by many signalling processes at the plasma membrane (PM), thereby modulating key processes such as proliferation^10^, migration, differentiation and survival^11, 12^.

Integrins, as the key mediators of cell adhesion, not only facilitate the mechanical anchoring of cells to extracellular supports but also originate the important ability of cells to sense the mechanical properties of their surrounding. Intriguingly, this mechanical information is directly transmitted via a continuous molecular connections between focal adhesions and chromatin rather than a signalling cascade of soluble messengers^13, 14^. In more detail, changes in the microenvironment are detected and transferred via actin and nuclear envelope proteins (nesprin-1 and 2, SUN 1 and 2) into the nucleus, leading to a reorganization of the nuclear lamina^15, 16^, the activation of transcription factors^17^ and to a change in the mechanical properties of the nucleus itself^18^. With Lamin as an indicator of stiffness perception and signalling to the nucleus it was shown that a cellular environment with a low stiffness leads to a soft nucleus, whereas the stiffer supports yields a stiff nucleus^18, 19^. Hence, integrins bring the culture conditions and chromatin organization into a direct molecular connection, with the result that the mechanical properties of the ECM are mirrored by the nucleus with the result of a mechanically balanced ECM-nucleus connection^15^.

With this connection in mind, it becomes apparent that any treatment of cells with the nucleus as the prime target needs to take this delicate balance into account. One such example is found in the treatment of cells, predominantly tumors, with ionizing radiation. While the prime reason of using radiation is to cause levels of DNA damage that ultimately lead to cell death, it was found that cells embedded in an ECM show a marked radioresistance towards ionizing radiation (IR) in comparison to conventionally 2D cultured cells^20^. This effect, also known as “cell-adhesion-mediated-radio-resistance” (CAM-RR), tellingly shows that the true impact of radiation on cell survival has to be understood as a combination of the radiation's damaging effect on DNA as well as its disturbing effect on the balanced ECM-nucleous connection. Along those lines, CAM-RR was linked (i) to ECM-binding integrins containing the β1 subunit and (ii) to the chromatin structure that differs between cells cultured on stiff surfaces versus cells grown on soft planar supports or under 3D culture conditions^21^. Namely, the presence of a higher fraction of heterochromatin in 3D cultured cells was shown to correspond to a decreased amount of residual DNA double strand breaks (DSB) after X-ray irradiation^22^. As (i) a higher amount of heterochromatin protects the DNA against DSB induction and (ii) a forced enrichment of euchromatin leads to hypersensitive DNA-damaging not only residual but also prompt DSBs may be reduced in 3D cells^21, 23, 24^. While integrins as the key mediators of a cell’s interaction with the ECM are clearly involved in this culture-condition dependent effect, mainly the players acting downstream of integrin signalling have been thoroughly investigated (ILK, FAK, JNK1, Akt1, PINCH1, HDAC)^25–28^. The membrane located effects of ionizing radiation on β1 integrins, involving the formation, dynamics and maintenance of integrin clusters to form focal adhesions (FAs) at the plasma membrane (PM), have so far not been in the focus of a detailed study, neglecting the possibility of the integrin distribution itself as a potential drug target for radiosensitization.

We therefore aimed to catch the very origin of the signalling that eventually leads to the changes that make cells radio-resistant in 3D environments, by following the plasma membrane located nanoscale organization of β1 integrins in response to both X-irradiation and culture conditions. For this, we optimized a collagen I based 3D cell culture system to be compatible with single molecule microscopy^29^ and used nuclear lamin organization to select for cells with a balanced ECM-nucleus connection.

To investigate the effects of IR on the integrin signalling we focused on the integrin clustering as well as on quantifiable parameters such as cluster density or number of integrins per cluster of 2D and 3D cultured cells. Our results show that (i) physiologically conditions lead to a different organization of β1 integrins and important downstream partner per se and (ii) X-ray irradiation leads to a nanoscale distribution of integrin β1 of 2D cultured cells at much lower dosages, as compared to cells cultured in our 3D cell culture system. We also show that integrins containing the β3 or αv subunit are affected differently by IR in dependence of the cell culture conditions. These results serve as an important entry point to characterize the membrane located events behind CAM-RR and may lead to the use of the integrin distribution as a potential drug target for radiosensitization.

## Results

### A 3D cell culture system tailored for single molecule microscopy and X-irradiation

**Figure 1:**
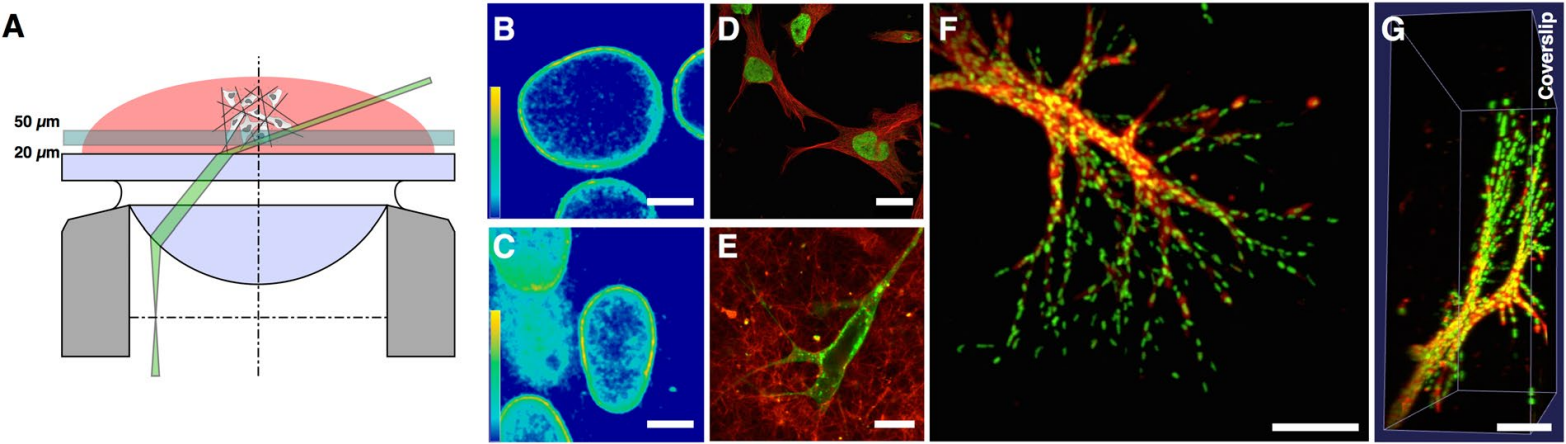
3D cell culture conditions have a strong impact on intranuclear lamin distribution and overall morphology of MEF cells. (**A**) 3D cell culture system for single molecule microscopy and irradiation experiments: Cells embedded in the collagen-hydrogel are placed on a coverslip. A thin, highly inclined laminar optical sheet (HILO) is used to illuminate a subfraction of fluorescently labeled primary integrin antibodies of cells cultured in 3D in a distance of of 20–50 µm to the coverslip. By combining STORM and HILO a sufficient imaging contrast is obtained to detect single molecule signals in 3D cultured cells. (**B**) Heat map of a Lamin A/C immunostaining of 2D versus (**C**) 3D cultured cells. Scale bar is 5 µm, respectively. (**D**) A β-Tubulin (red) and H2A (green) immunostaining visualize the cytoskeleton and the nucleus of 2D cultured MEF cells. Scale bar is 25 µm. (**E**) CMO (green) staining of the PM of a 3D cultured MEF cell cultured in a collagen hydrogel (red). Scale bar is 10 µm. (**F**) MEF cells after a week-long 3D culture (maximum projection of a 3D confocal stack). (**G**) 3D volume rendering of (**F**).

To investigate the effects of IR on the nanoscale organization of integrins with single molecule precision, we first had to optimize and characterize our approach to culture and image cells in a 3D collagen I based hydrogel, in order to meet the requirements set by the cells, the irradiation protocol, and the microscopy method. To achieve meaningful single molecule recordings and X-irradiation treatments, only cells within a distance of 20–50 μm to the coverslip were chosen for measurements. Within this range, reasonable contrast and image quality was achieved to detect single molecule signals with a localization precision of around 30 nm^29^. Also, at this distance, the glass doubling effect for radiation experiments^30^ still applies, allowing us to apply the same irradiation doses to cells cultured under 2D and 3D conditions for our comparative analysis (Fig. 1A).

In addition, cells within this range were completely surrounded by the collagen matrix^31^. More important from a mechanobiological point of view and the main rationale behind culturing cells in 3D hydrogels is, however, to provide cells an environment of low and isometric stiffness. As we were not able to measure the elastic modulus within a collagen hydrogel at the required position, we made use of the cells mechanosensing capabilities. For that we measured the nuclear distribution of lamin A/C in 2D and 3D cultured mouse embryonal fibroblast (MEF) cells, as this protein is known to change with the stiffness of the extracellular environment^18^. As expected, the distribution of lamin A/C to the nuclear envelope versus an intranuclear location was found to be much stronger in 2D than 3D cultured cells (Fig. 1B and C). Line profiles (see Supplementary Figure 1) reveal a higher level of non-nuclear envelope lamin A/C in cell cultured in 2D (~37%) versus 3D (~58%).

Hence, with lamin A/C as a read-out for nuclear stiffness perception, we can show that cells grown under our 3D conditions perceive a stiffness environment, that is markedly different from the planar rigid environment of a 2D culture, despite the low proximity to the coverslip of only 20–50 µm.

Furthermore, the drastically different morphology of MEF cells, as used throughout this study, was greatly affected by the 3D culture conditions (Fig. 1), both on the level of individual cells and multicellular arrangements. While 2D cultured MEF cells show the familiar flat morphology, MEF cells cultured in our 3D collagen system spread in all spatial directions (Fig. 1D–G). After a week-long culture a well ordered multicellular organization developed (Fig. 1F and G). Next to the results for intranuclear lamin A/C distribution, these morphological differences are an additional indication that our 3D culture system provides a microenvironment properties that are markedly different to the culture of MEF cells on planar rigid surfaces.

### The culture condition has a strong impact on the nanoscale distribution of integrins

While individual membrane proteins of cells cultured on coverslips can be imaged using TIRF microscopy^32^, 3D cultured cells are not amenable to this method^29^. To record and detect the location of single proteins in cells cultured in 3D collagen I hydrogels, we combined HILO illumination^33^ with STORM measurements^34^. With this approach, an axially confined illumination of cells distant to the coverslip is achieved by focusing the excitation laser off-center into the the back focal plane of a high NA objective. The beam refracts at the coverslip-water-interface and propagates into the 3D cell culture at a shallow angle to the coverslip, while also crossing the focal plane of the objective. The result is an axially confined illumination zone and axially and laterally confined detection zone with - in our case - a lateral diameter of typically 30 µm and a thickness (i.e. axial extension) of 1.2 µm. With this illumination scheme only a subfraction of STORM capable Alexa dyes (Alexa-488) linked to the respective antibody were activated, excited and detected allowing for a contrast sufficient to robustly detect individual antibodies and localize their signal with a precision of typically 30 nm. Single proteins in 3D cultured cells could thus be localized with a similar precision as in 2D cultured cells in our comparative analysis.

**Figure 2:**
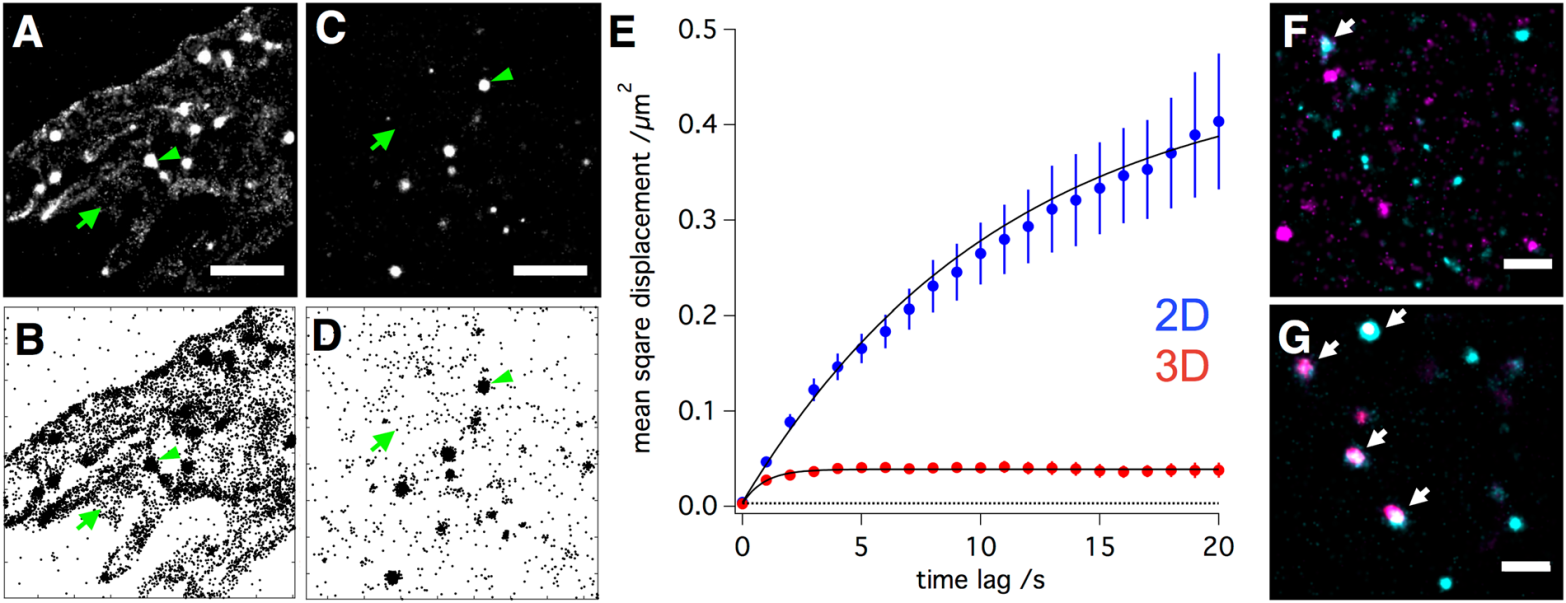
2D versus 3D cell culture conditions have a strong impact on the nanoscale distribution of integrins. (**A**) Normalised gaussian and (**B**) Scatter plot visualization of single molecule localization data obtained through a live-cell immunostaining of 2D cultured cells with a directly labeled integrin β1 antibody. Scale bar is 2 µm. (**C** and **D**) Corresponding data of a live-cell integrin β1 immunostaining of 3D cultured cells. A much higher fraction of integrins in 2D cultured cell is not part of a cluster as compared to 3D cultured cells (immobile integrin clusters = green arrowheads, individual mobile integrins = green arrows). Single molecule tracking of live cell antibody stained β1 integrins confirms this visual observation. Whereas the mean square displacement plot of β1 integrin mobility measured in 3D cultured cells shows a strong confinement (fast saturation of the curve) and low mobility (low slope, Fig. 2E, red), β1 integrins in 2D culture cells are, on average, much faster and far less confined in their mobility (Fig. 2E, blue). Fitting both plots to a model for confined diffusion reveals that β1 integrins in 2D and 3D cultured cells are confined in their mobility to an area of 1.2 ± 0,045 and 0.33 ± 0,005 µm^2^ with diffusion coefficients of 0,02 ± 0,0005 and 0,002 ± 0,0001 µm^2^/s, respectively. The dotted line represents the lower limit of the detectable square displacement (in our case 4× (28 nm)^2^ = 0,003136 µm^2^). (**F**) Colocalization of β1 (cyan) and β3 (magenta) integrins in 2D and (G) 3D cultured cells. Scale bar is 1 µm. Arrows indicate integrin-subunit colocalization (white) of β1 (cyan) and β3 (magenta) integrin subunits.

**Figure 3:**
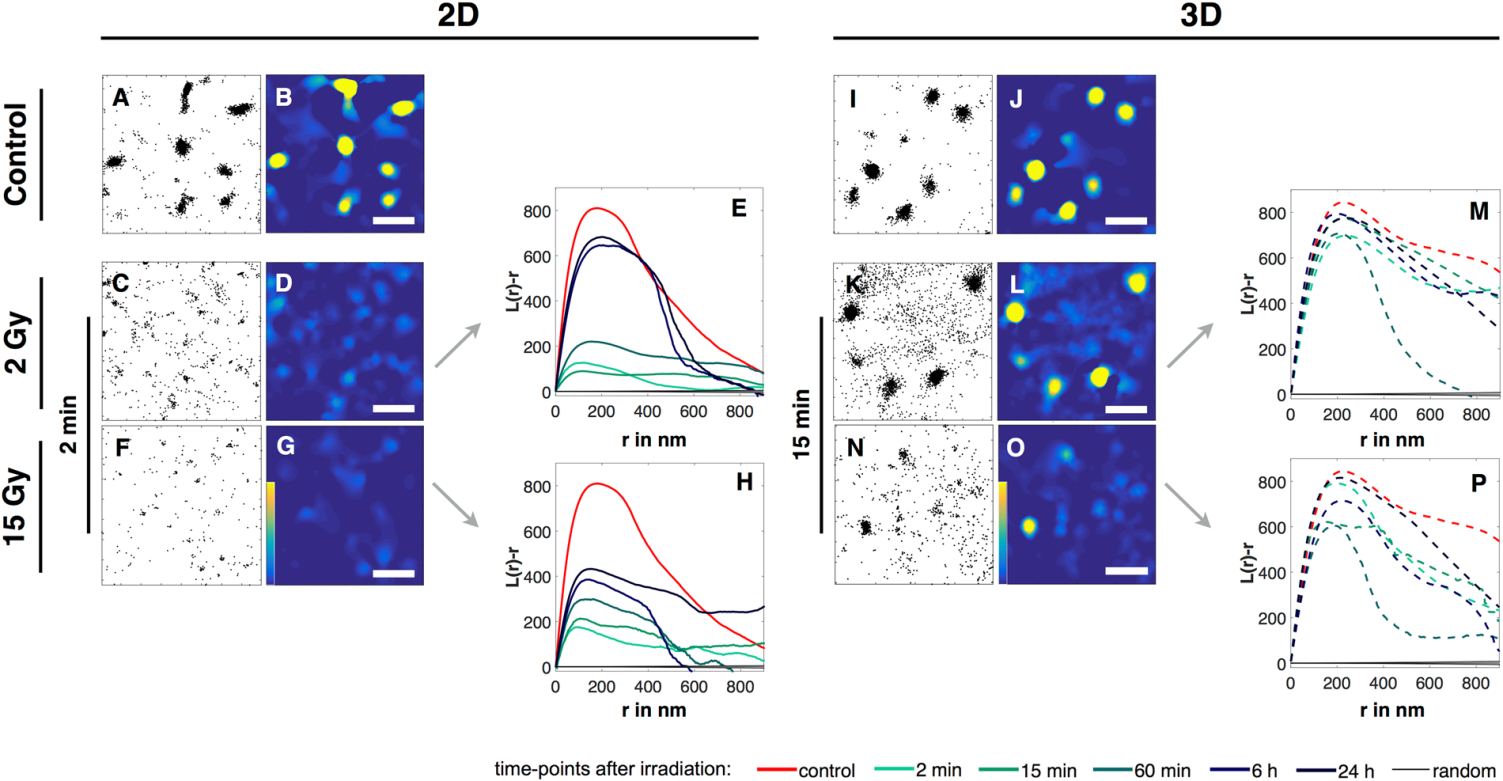
Effects of IR on the nanoscale distribution and organization of β1 integrins of 2D and 3D cultured MEF cells. Single molecule localization data of integrin β1 immunostainings obtained from fixed cells 2 min (2D) and 15 min (3D) after IR, as well as unirradiated controls are shown. Scatter plots show all detected β1 integrin localizations (**A**,**C**,**F** and **I**,**K**,**N**), while the corresponding heat maps (**B**,**D**,**G** and **J**,**L**,**O**) visualize unclustered (dark blue) and clustered (yellow) regions; scale bas are 1 µm. (**G**,**H** and **O**,**P**) Statistical analyses of all β1 integrin localizations found in all recorded cells of a given culture condition and irradiation dose are shown as H-Plots of Ripley’s K function, in which the peak heights (L(r)−r) represent the degree of clustering and its position the most frequent cluster size (r (in nm)). Shown are the distributions of β1 integrins as found for non irradiated, 2D cultured cells (**A**,**B**) for 2D cultured cells irradiated with 2 Gy (**C**,**D**,**E**), and for 2D cultured cells irradiated with 15 Gy, fixed 2 min after IR (**F**,**G**,**H**), respectively. The corresponding H-Plots (**E** and **H**) show results for control (red) and cells irradiated with 2 Gy fixed 2 min (turquoise), 15 min (light green), 1 h (green), 6 h (blue) and 24 h (dark blue) after IR. In addition, the analysis of a random distribution of localizations containing the same number of signals as the control is shown in black. (**I**–**P**) Corresponding data of 3D cultured cells, but fixed 15 min after IR. Heat maps of all remaining conditions (IR dose and time points after IR) are summarized in Figs S5 and S6.

With this technique we observed clear differences in the nanoscale distribution and mobility of β1 integrins in living cells (Fig. 2A–D). A live cell antibody staining revealed (by simple visual inspection of live recordings; data not shown) for 2D as well as 3D cultured cells that integrins are predominantly localized in non-mobile clusters (green arrowheads), a finding also reported by Rossier *et al*.^35^ for 2D cells. In addition, a significant fraction of β1 integrins was found to be unclustered and highly mobile in 2D but not in 3D cultured cells. This mobile fraction is best visualized in a scatterplot of all localization for the 2D culture (Fig. 2B) and 3D culture case (Fig. 2D), in which the single and separated pixels (green arrows) represent the mobile integrins. While in 2D around 25% of all detected integrins fall in this mobile category, in 3D, only very few integrins are not part of a cluster (10%). Single molecule tracking of live cell antibody stained β1 integrins confirms this visual observation. Whereas the mean square displacement plot of β1 integrin mobility measured in 3D cultured cells shows a strong confinement (fast saturation of the curve) and low mobility (low slope, Fig. 2E, red), β1 integrins in 2D culture cells are, on average, much faster and far less confined in their mobility (Fig. 2E, blue). Fitting both plots to a model for confined diffusion reveals that β1 integrins in 2D and 3D cultured cells are confined in their mobility to an area of 1.2 ± 0,045 and 0.33 ± 0,005 µm^2^ and display a mean mobility with diffusion coefficients of 0,02 ± 0,0005 and 0,002 ± 0,0001 µm^2^/s, respectively.

Our live cell single molecule measurements show that cells cultured in 3D achieve a much more defined organization of integrins in clusters. In addition, they also reveal a much lower separation of integrin subtypes into separate clusters (Figs 2F, G and S2). By staining different ECM-binding integrin combinations, namely αvβ1, αvβ3, and β1β3, we assessed the colocalization of integrins on cells fixed with a protocol optimized for membranes to avoid antibody-induced clustering of incompletely immobilized membrane proteins^36^. Not only the colocalization of the integrin subunits αv with β1 and αv with β3 was increased if cells are surrounded by the ECM (S2A,B), but even the integrins β1 and β3 were found to co-cluster in the PM of 3D (Fig. 2F) but not 2D cultured cells (Fig. 2G).

Taken together, 3D cultured cells showed a far lower distribution of integrins into separate clusters, but rather combine integrin heterodimers with similar function - here to bind the ECM - within the same clusters, i.e. focal adhesions. Virtually none of the integrins in 3D cultured cells were not organized in clusters. Thus, integrin clusters of 3D cultured cells were clearly found to be more defined and to contain more integrin subtypes.

### IR differently affects the nanoscale distribution and organization of β1 integrins in dependence of the culture conditions

To investigate the membrane-located events in response to IR, 2D and 3D cultured cells were X-irradiated with different doses, fixed at specific time points after the irradiation, and stained for endogenous β1 integrins. Due to the rapid and complete immobilization of the fixation protocol^36^, the following results represent snapshots of the dynamic and continuous process of protein cluster formation and degradation and thus show the current balance between these competing processes for the respective time point of fixation.

The effects of IR on the distribution and organization of β1 integrins can directly be recognized by a visual inspection of the single molecule localization results, where each dot represents an individual detection (Fig. 3A and I). Measurements on untreated 2D and 3D cultured cells reveal that β1 integrins are organized as clusters with a radius of ~200 nm and show a comparable degree of clustering under both 2D and 3D culture conditions (see S3). Upon IR, however, this picture changes in clear dependence on said culture conditions (Fig. 3C,F and K,N).

**Figure 4:**
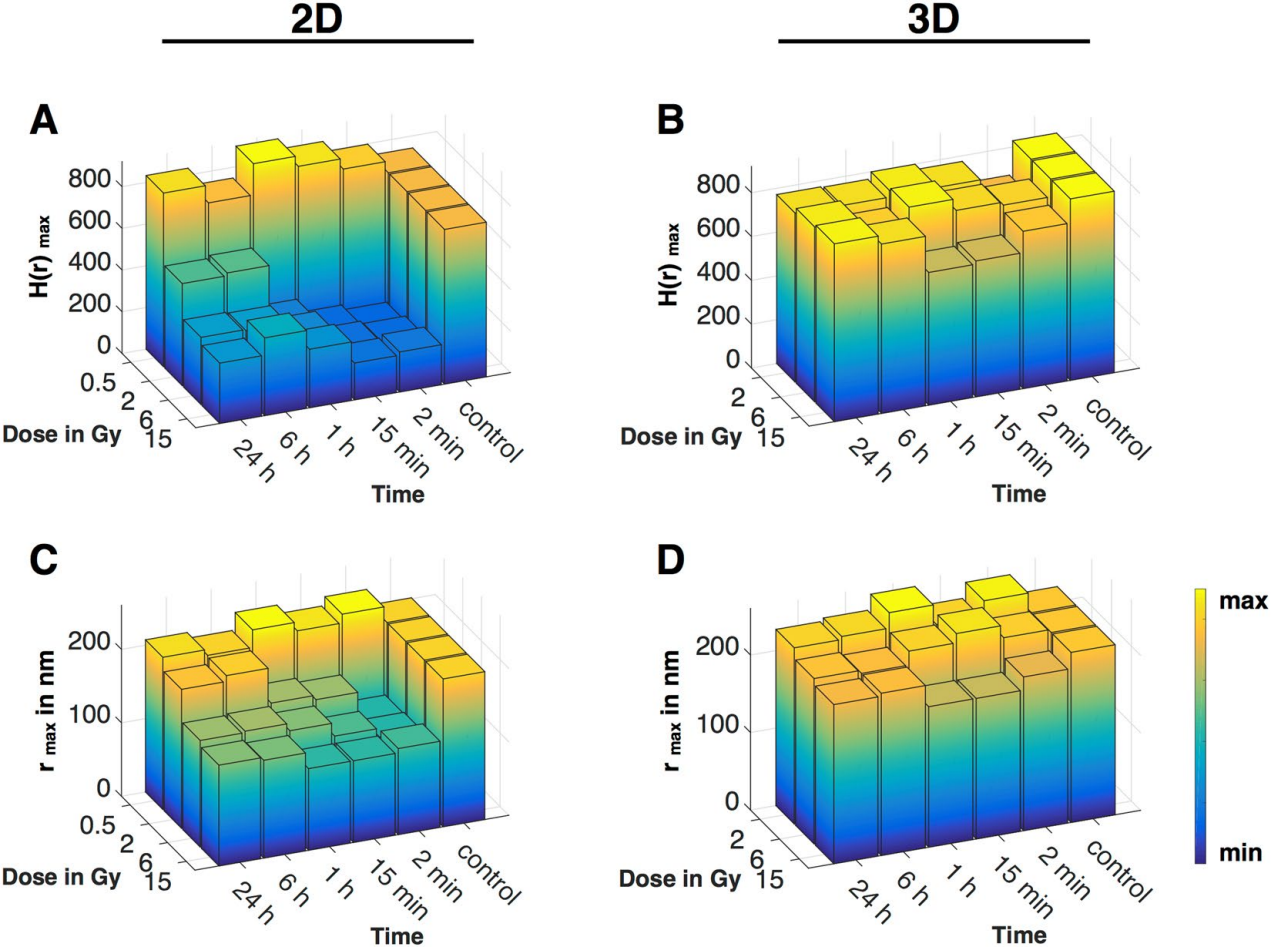
Effects of IR on the cluster density (H (r) max) and the cluster radius (r max in nm) of β1 integrins of 2D and 3D cultured MEF cells. Bar plots of medians of H (r) max and r max obtained from H-Plots of Ripley’s K function analysed datasets (S6), visualised as a combination of bar plot and heat map (low value in blue, high value in yellow). For 2D cells N = 2 and n = 20, for 3D cells N = 2 and n = 10. Controls were pooled, therefore for 2D cells N = 8, n = 80 and for 3D cells N = 6, n = 30. 2D cells were irradiated with 0.5, 2, 6 and 15 Gy, 3D cells were irradiated with 2, 6 and 15 Gy. Cells were fixed and stained as controls, 2 min, 15 min, 1 h, 6 h and 24 h after IR. (**A**) Plot of H max of 2D cultured cells. (**B**) Plot of r max of 2D cultured cells. (**C**) Plot of H max of 3D cultured cells. (**D**) Plot of r max of 3D cultured cells. For a better visualisation significances are displayed in S6.

Following visual inspection, a cluster analysis using Ripley’s K function helps to put the visual impression on quantitative grounds. In brief, the function computes the average number of signals that fall within defined radii of each detected signal. Plotting this number (L(r)−r)) versus the respective radii yields a distribution (H-plot) whose maximum represents the most prominent cluster formation in the dataset. The height of the first local maximum (H(r)max) gives a measure of the degree of clustering and its position the radius of the most frequent clusters^37–39^ (For further informations see S4). In addition, 2D plots of the L(r)−r values are represented as heatmaps to visualize clustered regions. Taken together, clusters, i.e. regions with a higher density of signals, are visualized as yellow regions in the heat maps (Figs 3B,D,G,J,L,O and S5 and S6) and the degree of clustering shows as the peak height in Fig. 3E,H and M,P. A summary of all maximum values for the degree of clustering (H(r)max) and the corresponding values for the cluster radii is shown in Fig. 4 for all X-irradiation doses, time points after irradiation and culture conditions.

2D cultured cells were irradiated with 0.5, 2, 6 and 15 Gy. While an irradiation with 0.5 Gy does not affect integrin clusters we could show that a dose of 2 Gy is sufficient to break the clustering of integrins directly after radiation (2 min) and that an irradiation with 6 and 15 Gy leads to an identical decrease in clustering (****p ≤ 0.0001 for 2, 6 and 15 Gy) (Figs 3D,G and S5). The kinetics of cluster regeneration was found to strongly depend on the initial dose, as clusters of cells irradiated with 2 Gy regenerate faster as clusters of cells irradiated with 6 or 15 Gy. Corresponding H-plots of Ripley’s K function and summarized bar plots of the degree of clustering reveal that the clustering of integrins of 2D cultured cells irradiated with 2 Gy starts to regenerate 1 h after IR and nearly returns to full recovery 6 h after IR (Figs 3E and 4A). After an irradiation with 15 Gy clusters also start to regenerate 1 h after IR, but even 24 h after IR recovery remains incomplete (Figs 3H and 4A). As H max and the cluster radius r max correlate, the same effects were expected and detected. While an irradiation leads to a significant decrease (****p ≤ 0.0001) of cluster radii, 2 Gy irradiated cells regenerated completely, whereas 6 and 15 Gy irradiated cells do not (Fig. 4C). An irradiation with 0.5 Gy did not show a significant effect on the cluster radius.

For cells cultured in 3D, the effects of ionizing radiation differ greatly. In contrast to 2D cultured cells, irradiation doses of 2 and 6 Gy had no discernible effect on the organization of β1 integrins in clusters. Only at a dosage level of 15 Gy slight, but significant changes in the clustering (*p ≤ 0.05) and their radii became apparent (Figs 3L,O and 4B,D and S7). Moreover, not only the dosages required to affect the clustering of β1 integrins are significantly higher in 3D cultured cells, also the kinetics of the process differ. In contrast to 2D cultured cells, an effect on β1 clustering started to become discernible only 15 min after an IR of 15 Gy with an almost complete recovery after 6 h and a full recovery after 24 h (Fig. 3P).

Taken together, these results clearly reveal a strong dependance of the nanoscale organization of β1 integrins on the culture conditions with 3D cultured cells being far more resistant to IR in maintaining the clustered organization of the adhesion receptor and showing a much faster recovery to do so after high IR doses.

The distinct and, at the same time, rapid effects of IR on membrane organization suggest that rather than DNA damage, more immediate effectors are responsible for the swift response. X-irradiation is always accompanied by the generation of reactive oxygen species (ROS) which leads to the damage of many structural and functional molecules^40^. Hence, to examine the role of ROS, 2D cultured cells were treated with 100 µM H_2_O_2_ and 3D cultured cells were treated with 500 mM H_2_O_2_ prior to fixation and integrin β1 staining. In response to the treatment, the degree of clustering as well as cluster radii significantly decreased 2 min for 2D and 15 min for 3D cells after the treatment (Fig. S8) reaching the same level as irradiated cells. Thus, as the kind, extent and timing of H_2_O_2_ are similar to those observed after IR, radiation produced ROS are likely the main, if not the sole cause for the disintegration of integrin clusters.

While we are aware that 500 mM is an unnaturally high concentration of H_2_O_2_, lower concentrations did not cause an discernible effect on the clustering of integrins in 3D cultured cells. Of note, however, the all 3D cultured cells did survive this treatment without major changes in morphology (data not shown).

### Detailed cluster analysis reveals distinct differences in the effects of IR on the clustering and the cluster size of β1 integrins

To gain further insight into the membrane located events in response to ionizing radiation and in dependence of the culture conditions, we sought to extract more quantitative values from the cluster analysis of our single molecule localization data. For that, we defined a common threshold value that defines a cluster to have a higher signal density than the mean of all untreated cells.

**Figure 5:**
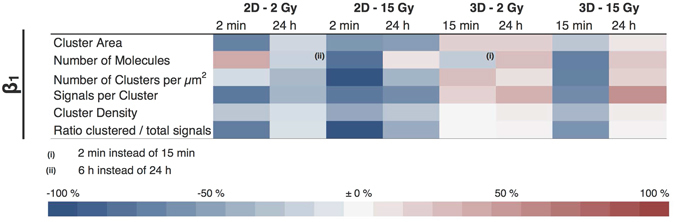
Percentual change of various parameter after IR of β1 integrins of 2D and 3D cultured MEF cells.

Using this value, heat maps were converted into binary cluster maps, from which parameters like cluster area and number of clusters per μm^2^ were directly obtained. Masking the original localization data with these binary maps yielded additional values like signals per cluster, signals per cluster area (i.e. cluster density) as well as total number of signals and the ratio of clustered vs total number of molecules. To simplify inspection of the results, we summarized them in a heat map table (Fig. 5), where negative changes are assigned to a blue and positive to a red colour intensity. Changes were determined 2 min and 24 h for 2D, and 15 min and 24 h after irradiation for 3D cultured cells.

In detail, cells cultured in 2D irradiated with 2 Gy showed a significant decrease in the cluster area, the signals of molecules per cluster and in the ratio of clustered versus total number of molecules. The total number of molecules increased, while the number of clusters and the cluster density were only slightly affected. 24 h post-irradiation, almost all parameters returned to their pre-irradiation values. At 15 Gy, except for the total number of signals, a similar trend but more drastic changes were found. The most significant decreases are seen for the the number of clusters and the ratio of clustered vs unclustered molecules. While the number of molecules exceed the initial value, most parameters did recover but failed to completely return to the initial values. Only the cluster area did not show any recovery.

In contrast, cells cultured in 3D irradiated with 2 Gy showed an entirely different reaction towards IR. Except for the number of molecules, where only a slight decrease was detected, all other parameter showed none or slight, but never significant increases. 3D cells irradiated with 15 Gy showed a decrease in all parameters 15 min after IR, but all of them returned to or exceeded their initial value. Details for all values and test for significance are summarized in S9–11

### β3 and αv integrins are differently affected by IR than β1 integrins

To investigate whether IR has an effect on integrins containing αv or β3 the same set of comparative experiments were performed and summarized in S12–18, as well as in Supplementary Table 1. Both integrin subunits showed, in comparison to β1, an entirely different reaction towards IR, which, on its own, strengthens the significance of our findings for the latter. While integrin heterodimers containing the β3 subunit barely show any changes to the various treatments and culture conditions, integrins containing a αv subunit revealed intermediate results. As αv dimerizes with both β subunits and the resulting integrin heterodimers have different cellular functions^41^, namely cell adhesion (αvβ1) and migration (αvβ3)^42^, this comes as no surprise. It does, however, nicely demonstrate the capabilities of single molecule over ensemble measurements. As the behaviour of all integrin subunits are recorded individually and all interactions with their partners are recognized individually, the behaviour of αv, which is here presented as an average, could as well be dissected into to its interaction with only β1 or β3. A more detailed presentation and discussion of the αv and β3 data can be found in the Supplementary.

### FAK phosphorylation status markedly differs before and after irradiation in dependence of the culture conditions

**Figure 6:**
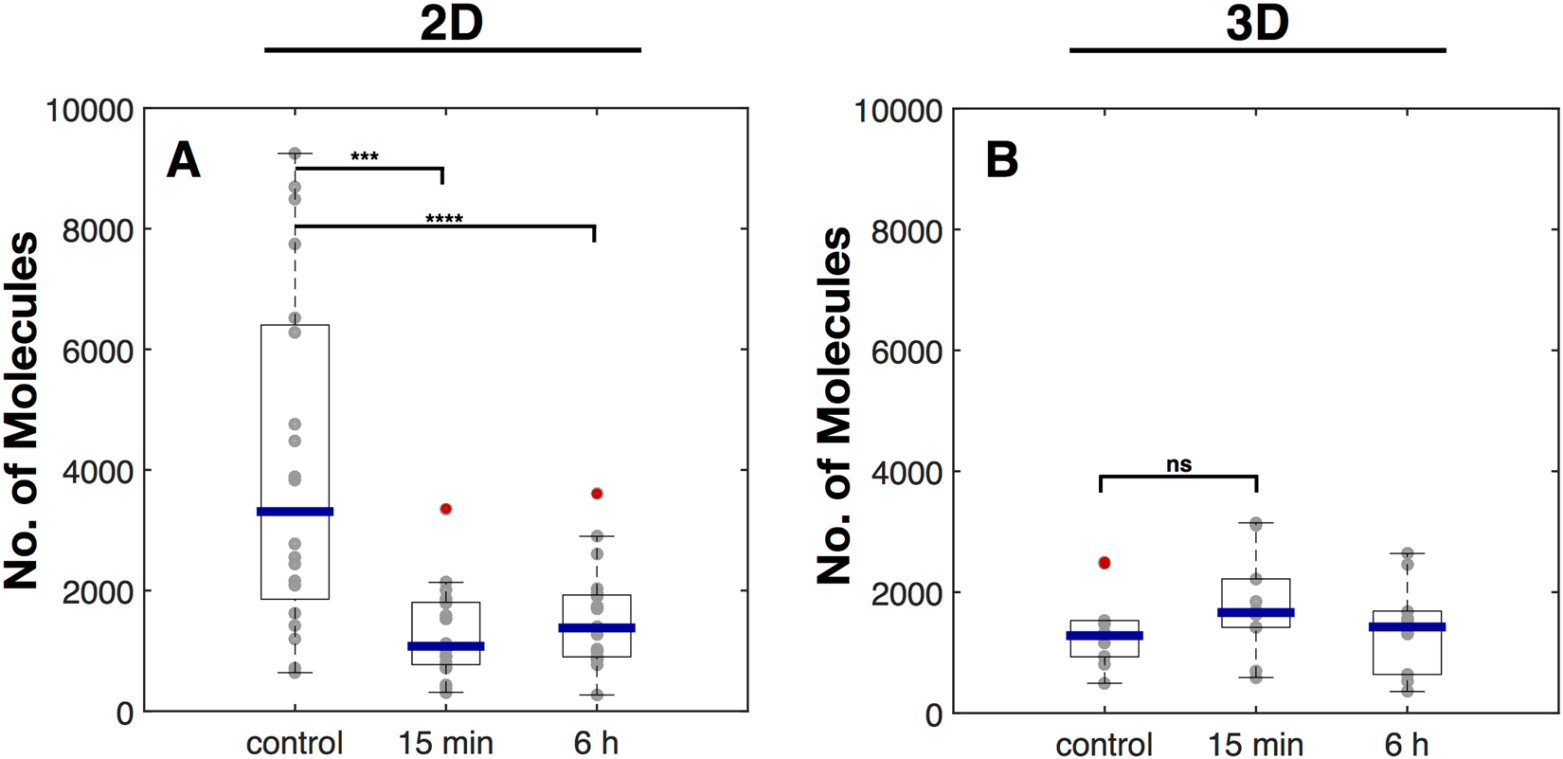
Effects of IR on pFAK of 2D and 3D cultured MEF cells. Box plots of the number of molecules per 4 × 4 µm ROI of cells cultured in 2D (**A**) and 3D (**B**). Cells were irradiated with 6 Gy and were fixed 15 min and 6 h after irradiation. For 2D cells N = 2 and n = 20, for 3D cells N = 2 and n = 10. Statistical analysis was performed with an ordinary one-way ANOVA. ***p ≤ 0.001 and ****p ≤ 0.0001. NS, not significantly different. If not further noted no significance was detected.

Through our detailed analysis on integrins, we have clearly shown how their nanoscale distribution and organization changes in response to IR and culture conditions. However, for these differences to have an effect on cellular reactions, survival and ultimately CAM-RR, signalling from the differently organized and - upon IR - disintegrated integrin clusters need to change as well. We therefore assessed the IR and cell culture dependence of the integrin’s immediate downstream partner, the focal adhesion kinase (FAK), and quantified the amount of its phosphorylated, i.e. activated from, pFAK (Fig. 6). While the number of pFAK in untreated cells was found to be about twice as high in 2D versus 3D cultured cells, the amount after an irradiation with 6 Gy was nearly identical for both culture conditions and did not recover within 6 h. pFAK levels of 3D cultured cells were not affected by an irradiation with 6 Gy.

These findings are in line with our observation that 2D cultured cells are unable to maintain integrins in well defined clusters and tend to separate different heterodimers into distinct clusters rather than combining them as the 3D cultured cells. Hence, a possible interpretation for the pFAK results is, that integrin signalling in 2D cultured cells is able to initiate the signalling cascade, but may be ineffective further downstream. In consequence, a higher pool of pFAK builds up, but is likely to be ineffective to properly propagate the signal in 2D cultured cells, whereas this is not the case in 3D cultured cells.

## Discussion

Studies on the cellular effects of IR are generally focused on DNA damage, its subsequent repair, checkpoint activities and, ultimately, cell survival^43^. With this focus, it was identified that cell adhesion and, in particular, cellular growth within the 3D environment of extracellular matrix proteins renders cells resistant to higher doses of ionizing radiation than cells grown on planar rigid supports. This resistance was reflected in a lower number of residual DNA double strand breaks (DSBs) and a higher survival rate and was termed cell adhesion mediated radioresistance (CAM-RR)^20, 23^. While the origin of this culture-condition dependent resistance is clearly located at the integrins as the key proteins that sense and report the properties of the extracellular environment, so far mainly the players acting downstream of integrin signalling have been thoroughly investigated (ILK, FAK, JNK1, Akt1, PINCH1, HDAC)^25–28^. To fill this gap, we focused in our study on the immediate and rapid effects of IR on the nanoscale organization of the plasma membrane of cells grown under classical 2D as well as 3D culture conditions. To that end, we characterized the impact of IR on the formation, dynamics and maintenance of integrin clusters to form focal adhesions (FAs) at the plasma membrane (PM) using single molecule localization microscopy (SMLM). Our achievement to apply the virtues of SMLM to investigations on nanoscale changes of membrane protein distributions in cells grown under both conditions with comparable accuracy, allows us to draw detailed comparative conclusions regarding the influence of a cell’s environment on the membrane located events behind CAM-RR.

We found that already the culture conditions in absence of any irradiation cause a marked difference in the ability of cells to maintain a well organized membrane. While 3D cultured cells keep nearly all integrins in a clustered organization, contain more β1 integrins per cluster and even keep different integrins within the same clusters (αvβ1, αvβ3 and β1β3), 2D cultured cells are unable to maintain this level of organization for these aspects. A significant portion of β1 integrins was found to freely diffuse in the membrane, rendering them unable to take part in signalling. In addition, integrin subtypes were found to segregate into separate clusters rather than colocalize within the same clusters as in the 3D case. Intriguingly, this less defined clustering was accompanied by a significantly higher number of phosphorylated FAK (pFAK) in 2D versus 3D cells, indicating an overall impaired signalling efficiency at planar culture conditions. Taken together, mechanisms to keep a well defined integrin cluster organization are acting efficiently in 3D but not 2D cultured cells.

This inability of cells to maintain a well defined cluster status for integrins and an effective integrin signalling already points to a lower tolerance for additional stressors. In fact, we found that even low doses of IR are sufficient in 2D to completely abolish the cells ability to maintain integrins clustered, while 3D cultured cells were not only able to maintain the clustered organization against much higher doses of X-irradiation but also showed a faster recovery for doses that actually did induce a mild effect (15 Gy).

In detail, already a 2 Gy dose caused the number of signals per cluster to decrease and the number of unclustered molecules to increase (i.e. the ratio of clustered to total signals to decrease). In other words, an irradiation with 2 Gy caused β1 integrins to leave the clusters but stay in the PM by lateral diffusion. An irradiation with 15 Gy, in turn, not only caused the signals per cluster but even the total number of signals to decrease. This implies that the higher dose, in addition to disintegrate clusters within the plane of the membrane, also induced endocytic retrieval of integrins and thus induced both lateral and axial membrane transport. In 3D culture cells, the same combination of axial and lateral membrane transport was taking place, however at much higher doses. An irradiation 15 Gy lead to a decrease in the clustering, the cluster radius and cluster area, as well as to an decrease in the total number of molecules. Hence, while the mechanism of the response to high doses of were the same for both 2D and 3D cultured cells, the kinetics and the severity differ significantly (Fig. 4).

Thus, as stated above, the membrane organization of β1 integrins in 2D is less defined and therefore more vulnerable to disturbance than in 3D cultured cells. Notably, however, as our results on β3 and αv integrins show, this behaviour is protein specific and does not apply to the nanoscale organization of the membrane in general. The impact of IR on integrin clustering are also directly reflected by the effects on integrin signalling. As 3D cells did not respond with any change in the number of pFAK, the high levels found in untreated 2D cells collapsed to a very low amount after X-irradiation, indicating a severe impairment of signalling and IR load. Hence, the ability to maintain integrin clusters against IR is a direct consequence of intact integrin signalling.

Using a treatment of cells with H_2_O_2_ we were able to recreate the effect of IR to break down β1 integrin clusters in 2D (100 µm H_2_O_2_) and 3D (500 mM H_2_O_2_) cultured cells, demonstrating that ROS produced by IR are potentially the prime reason behind the observed effects of X-irradiation. While an identical treatment with H_2_O_2_ on 3D cultured cells would have been desired we were not able to produce an effect on the integrin clustering with low concentrations of H_2_O_2_. This may be explained by a very fast scavenging of ROS within 1–2 min in 3D cultured cells as it was reported for the generation of ROS in cells by gas plasma treatment^44^. As this method produces ROS in an abiotic way as does IR, we assume that 3D cultured cells have a high capacity to scavenge ROS.

But regardless, whether 3D cultured cells are able to scavenge ROS more efficiently or whether their membranes tolerate higher ROS levels, both accomplishments would simply be a direct result of the fact that the integrin signalling system of these cells is intact and tolerant to some degree of disturbance. This is reflected by our observations of (i) stable integrin clusters and (ii) stable levels of pFAK in presence or absence of IR and (iii) a different distribution of lamin A/C, which demonstrates the continuous signalling connection between the PM and the nucleus.

Our detailed view on the membrane located events involving integrin β1 and its downstream partner pFAK, allows us to conclude that CAM-RR starts at maintaining integrin clusters, as we show that only 3D cells possess and maintain integrins in a firm and defined organization of clusters and a signalling which, via FAK, eventually propagates all the way to the nucleus and impacts the distribution of the nuclear scaffold protein lamin A/C, which is known to influences the chromatin organization and even the stiffness of the nucleus itself^18, 19^. This connection from integrins to chromatin nicely links our results to the work of Cordes and coworkers who showed that the ratio of hetero to euchromatin changes with the cell culture condition, (1:1 for 2D, 2:1 for 3D cultured cells)^21^. Moreover, they were able to directly link the increased chromatin condensation with the ability of cells to survive after irradiation and the total number of DNA double strand breaks.

**Figure 7:**
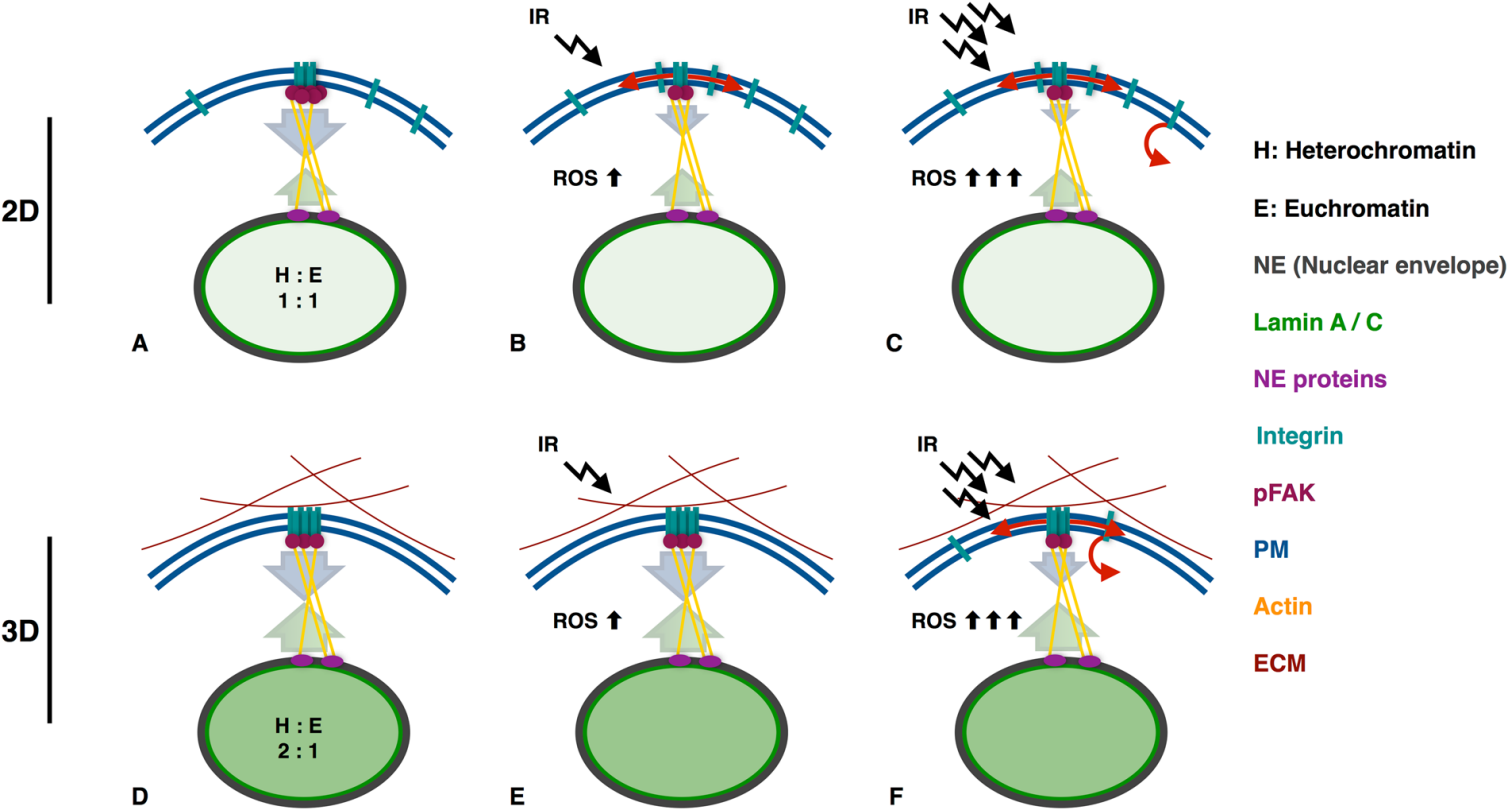
Mechano imbalance of 2D cells leads to their radio-sensitivity. (**A**) 2D cells show a different organisation of the nuclear matrix (Lamin A/C) and the chromatin condensation (ratio of hetero to euchromatin) if compared to 3D cells (**D**). The counterbalance of the nuclear force feedback between the integrin - cytoskeleton - nucleus link is imbalanced leading to a loose, dynamic integrin organisation in 2D cells (**A**). In 3D cells the mechanical link is well-balanced leading to firm and defined integrin clusters (**D**). An irradiation with a low dose (2 Gy) of 2D cells leads to integrin cluster disintegration by lateral diffusion and down regulation of downstream signals (pFAK) by up regulation of ROS. The imperfect force feedback combined with loose integrin clusters is responsible for the observed effects (**B**). As 3D cells exhibit a mechano-homeostasis, low doses can not effect integrin and pFAK signalling, although ROS is upregulated (**E**). (**C**) An irradiation with a high dose (15 Gy) exceeds the threshold for endocytosis leading not only to cluster disintegration by lateral diffusion but also by axial membrane transport. (**F**) 3D cells irradiated with a high dose (15 Gy) lead to a slight integrin cluster disintegration by a lateral and axial membrane transport. The equilibrium of the force feedback leads to effects that are shifted in their time dependence and in their intensity.

Taken together, we assume that the mechanical link connecting integrins with the nuclear lamina and therefore with the chromatin organization itself is differently balanced in cells cultured in 2D or 3D, leading to a different mechanosensitive homeostasis and to a different integrin cluster reaction upon irradiation. Our results involving the pFAK signalling support this assumption. pFAK signalling of unirradiated 2D cells is twice as intensive as detected for 3D cultured cells. This obvious imbalance of 2D cells leads to an incomplete nuclear force feedback and to a loose, dynamic integrin organization which is easily distributed. This leads to a integrin cluster disintegration and the loss of pFAK signalling at low dosages. The counterbalanced nuclear force feedback of 3D cells results in defined and firm integrin clusters. These clusters can only be disrupted at very high dosages resulting in a higher ROS level. This view is summarized in our model (Fig. 7).

## Conclusion

In conclusion, CAM-RR is based on an intact integrin signalling system connecting the PM with chromatin, which has its origin in the proper nanoscale organization of integrins. Only with this balanced system in place, the long term observations of CAM-RR (chromatin organization → lower amount of DSB → higher tolarance to IR → cell survival) are able to unfold. Consequently, it is reasonable to assume that if radioresistance relies on intact integrin clusters, an active disintegration of integrin clusters may in turn induce radiosensitivity - and probably also chemosensitivity, making the nanoscale distribution of integrins a potential drug target for radiosensitization. While integrin receptors have been in the focus as a drug target for a long time, almost all antagonists (antibodies, peptides or small molecules) so far targeted either the ligand-binding site or the ligand itself^45^. Based on our study, we propose in turn to target antagonists against the ability to form clusters. Along with this suggestion, however, microscopy capable to monitor the nanoscale distribution in cells cultured in meaningful 3D environments would need to become an integral part of the preclinical screening process.

## Materials and Methods

### Cell culture

MEF cells (mouse embryonic fibroblasts) were cultured in DMEM/Ham’s F-12 (1:1) (Biochrom, Berlin, Germany) supplemented with 10% FCS (Sigma-Aldrich, St. Louis, Missouri, USA) and 1% NEAA (Biochrom, Berlin, Germany) in a humidified chamber at 37 °C and 5% CO_2_. All cell cultures were prepared on round coverslips (Karl Hecht GmbH, Sondheim, Germany, NO. 1.5, Ø = 25 mm).

### 3D cell culture

MEF cells were cultured in 1.5 mg/ml collagen I hydrogels for 4 to 5 days prior to use. Functionalization of the coverslip surface with APTS is crucial for the immobilization of the hydrogels on the coverslip.

Coverslip coating with APTS ((3-Aminopropyl)triethoxysilane, Sigma-Aldrich, St. Louis, Missouri, USA). Coverslip were washed for 10 min in technical acetone (AppliChem, Darmstadt, Germany) and were dried afterwards. APTS solution was prepared freshly containing 2% (v/v) of APTS in acetone. 40 µl of the APTS solution was spined onto a dried, pre-cleaned coverslip using a spin coater. Coverslips were dried afterwards followed by two washing steps in H_2_O, each 10 min. Coverslips were dried and stored in the dark until use.

### Hydrogel preparation

Collagen I hydrogels were prepared similar to Vira *et al*.^46^. A 1.5 mg/ml collagen solution was prepared with 50% (v/v) of a 3 mg/ml rat-tail collagen I stock solution (Thermo Fischer Scientific, Waltham, Ma, USA), 7.5% (v/v) of 10x PBS (Thermo Fischer Scientific, Waltham, Ma, USA), 2.8% (v/v) of a 7.5% NaHCO3 (AppliChem, Darmstadt, Germany) solution and with 14.7% (v/v) of a cell suspension containing 5 × 10^3^ cells per µl. Cell suspension was obtained from one t25 flask of 80% confluent MEF cells. Cells were centrifuged after trypsin treatment and subsequently suspended in 1 ml media. 15 µl of the 1.5 mg/ml collagen solution was pipetted on a APTS coated coverslip. Hydrogels were incubated for 30 min at 37 °C and 5% CO_2_, afterwards fresh media was added and cells were cultured as described above.

### Radiation experiments

Irradiation was carried out using an Isovolt 160 Titan E (GE Sensing & Inspection Technologies, Alzenau, Germany) x-ray source. Cells were irradiated with a voltage of 90 kV and a current of 19 mA (for 0.5, 2 and 6 Gy) or 33.7 kV (for 15 Gy). Doses were delivered at a 30 cm source to probe distance with cell cultures placed on a 2 mm aluminum filtering plate with respect to the glass doubling factor^30^.

Cells were fixed at 2 and 15 min as well as at 1, 6 and 24 h after radiation with a protocol optimized for the fixation of membranes, to avoid antibody-induced clustering of incompletely immobilized membrane proteins^36^. In using 4% PFA (Carl Roth, Karlsruhe, Germany) supplemented with 0.2% glutaraldehyde (Serva Electrophoresis, Heidelberg, Germany) in PBS (Sigma-Aldrich, St. Louis, Missouri, USA), (pH 6.9) for 1 h at 4 °C. This protocol is optimized for the fixation of membranes. Controls were treated exactly as the irradiated cells, controls were fixed coincidently with the 2 min probes. Cells were washed once with PBS prior to fixation and three times after fixation followed by antibody labeling as described below.

### ROS Treatment

Cells were treated with H_2_O_2_ for 1 min (2D 100 µM, 3D 500 mM, Sigma-Aldrich, St. Louis, Missouri, USA)^47^ in HBSS buffer, afterwards they were washed 3 times with PBS and were incubated until fixation in HBSS (Biochrom, Berlin, Germany) buffer at 37 °C. Fixation with 4% PFA supplemented with 0.2% glutaraldehyde in PBS occurred 2 min for 2D and 15 min for 3D cells after treatment followed by antibody staining of β1 integrins.

### Stainings

#### Collagen staining

To stain collagen I the 3D cell cultures were incubated in a 20 µg/ml Erioglaucine disodium salt (E133, Sigma-Aldrich, St. Louis, Missouri, USA) solution for 10 min. Hydrogels were washed 3 times with PBS and imaged (Em: 633 nm, Ex: 750–750 nm).

#### Plasma membrane staining

For the detection of the plasma membrane CellMask Orange (Molecular Probes, Thermo Fisher Scientific, Waltham, Ma, USA) was used at a concentration of 0.5 µg/ml. Cells were incubated for 15 min in CellMask orange and imaged.

#### Antibody stainings

All integrin antibodies were purchased by Biozol Diagnostica (Eching, Germany). The following antibodies were used: anti CD29 Alexa 488 (integrin β1), anti CD61 Alexa 488 (integrin β3), anti CD51 (integrin αV) and anti-rat Alexa 647 (Biozol Diagnostica, Eching, Germany). All primary antibodies bind to extracellular integrin domains, therefore no permeabilization was needed. For all SMD measurements a 1:10 000 antibody dilution was used for 2D cells, for 3D cultured cells antibodies were diluted 1:5 000. For live cell measurements of β1 integrins cells were washed twice with PBS. Cells were incubated for 15 min in β1 integrin antibody solution in HBSS buffer. Afterwards cells were washed with PBS and were imaged directly. To analyse single molecules of fixed cells, cells were blocked after fixation for 1 h with a 1% BSA (AppliChem, Darmstadt, Germany) solution at 37 °C, washed with PBS 3 times and were incubated for 3 h at 4 °C in the desired antibody solution. For labeling αV with a secondary antibody or for colocalization experiments cells were blocked and stained once more as described before. Cells were washed 3 times afterwards and were imaged using STORM buffer. For SMD measurements of pFAK cells were stained and imaged as described for the αV antibody combination, in addition cells were permeabilized with 0.2% Triton-X100 (AppliChem, Darmstadt, Germany). pFAK antibody (Santa Cruz, Dallas, Texas, USA) was applied 1:5000, anti-rabbit Alexa 488 antibody (Thermo Fisher Scientific, Waltham, Ma, USA) was applied 1:10000. All primary antibodies were used for CLSM measurements as 1:100 dilutions, secondary antibodies were applied as dilutions of 1:200. Cells were fixed with 4% PFA for 1 h at 4 °C, washed 3 times with PBS. Prior to the staining cells were permeabilized with 0.2% Triton X-100 solution in PBS for 10 min and were blocked for 1 h with a 1% BSA solution at 37 °C and were washed with PBS 3 times. For the visualization of the cytoskeleton, the nucleus and Lamin A/C, cells were stained with primary antibodies for β-tubulin (Sigma-Aldrich, St. Louis, Missouri, USA), H2A (Sigma-Aldrich, St. Louis, Missouri, USA), LMNA (abcam, Cambridge, UK) and secondary antibodies labeled with Alexa 568 (anti-mouse, Thermo Fisher Scientific, Waltham, Ma, USA) or 488 (anti-rabbit, Thermo Fisher Scientific, Waltham, Ma, USA), each 3 h at 4 °C.

### Microscopy

#### Microscopy buffers

Live cell imaging was performed in HBSS buffer (Biochrom, Berlin, Germany). For single molecule detection of fixed cells a standard STORM buffer was used according to Dempsey *et al*.^48^ and van de Linde *et al*.^49^. 100 mM MEA (beta-mercaptoethylamine, pH 8.5, Sigma-Aldrich, St. Louis, Missouri, USA), 140 U catalase (Sigma-Aldrich, St. Louis, Missouri, USA, C3515) and 10 U glucose oxidase (Sigma-Aldrich, St. Louis, Missouri, USA, G0543) were added immediately before use into Tris-buffer (50 mM Tris, 10 mM NaCl (both AppliChem, Darmstadt, Germany), pH 8) supplemented with 10% (w/v) glucose (AppliChem, Darmstadt, Germany). Imaging was performed under oxygen exclusion conditions.

#### CLSM measurements

CLSM-measurements were performed using the Leica TCS SP5 II (Leica Microsystems, Mannheim, Germany) equipped with a 63 × 1.2 Water corr objective or with a 63 × 1.3–0.6 oil objective.

#### SMD measurements

Single molecule imaging was performed using a custom-built instrument: The outputs of four continuous-wave optically pumped semiconductor laserdiodes (OPSL, Coherent Inc., Santa Clara, CA, USA) with wavelengths of 405 nm (OBIS, 100 mW), 488 nm (Sapphire, 75 mW), 561 nm (Sapphire, 75 mW), and 640 nm (OBIS, 100 mW) were controlled by an acousto-optic tunable filter (AOTFnC-400.650-TN and MDSnC, AA Opto-Electronic, Orsay, France), coupled into a single mode fiber (kineFLEX™, Qioptiq, Excelitas Technologies Corp., Waltham, MA, USA), and cleaned by a quadband excitation filter (FF01–390/482/563/640–25, Semrock, Rochester, NY, USA).

For widefield illumination the beam exiting the fiber collimator was expanded 15-times and focused into the back focal plane (BFP) of a Nikon CFI Apo TIRF 100x objective (NA 1.49, WD 0.12 mm) via a quadband dichroic mirror (Di01-R405/488/561/635–25 × 36, Semrock, Rochester, NY, USA) to exit the objective as a collimated beam with a FWHM of ~42 µm. Objective and filters were mounted in a Nikon Ti-E stand (Nikon, Konan, Minato-ku, Tokyo, Japan) equipped with a Perfect Focus System (PFS). For HILO and TIRF imaging the focus in the BFP was moved off-center by controlling the position of a mirror with a single-axis stage M-126. DG controlled by a C-863 Mercury Servo Controller (Physik Instrumente (PI), Karlsruhe, Germany).

For standard widefield fluorescence illumination, light from a Prior Lumen 220 Pro metal halide lamp (Prior Scientific, Cambridge, UK) was filtered by singleband excitation filters (FF01–390/40–15, FF02–482/18–25, FF01–563/9–25, FF01–640/14–25, Semrock, Rochester, NY, USA) and coupled into the microscope via a liquid light guide using the second of two of the Nikon Microscope’s ‘stratum structure’ beam paths. Fluorescence emission was imaged onto an Andor iXon EM + DU-897 (back illuminated) EMCCD camera (Andor, Belfast, UK) using either Semrock quadband (FF01–446/523/600/677–25), dualband (FF01–523/610–25) or singleband emission filters (FF01–445/45–25, FF03–525/50–25, FF01–612/69–25, FF01–731/137–25, Semrock, Rochester, NY, USA). The AOTF and single-axis stage were controlled by a custom written virtual instrument (VI) for Labview (National Instruments, Austin, TX, USA) using a NI PCIe-6323 data acquisition (DAQ) board (National Instruments, Austin, TX, USA) and the fire signals from the camera as timing triggers. The open source software Micro-Manager 1.4^50^ was used for image acquisition.

### Image acquisition and data analysis

Editing of confocal images was performed using Fiji (version: 1.48t)^51^. Single molecule signals were detected and filtered using the ThunderStorm plugin^52^ for Fiji. For single particle tracking and mean square displacement analysis TrackMate^53^ was used. As a model for confined diffusion, the following formula was used to fit the mean square displacement plots (see ref. 54 for further details on single molecule tracking analysis).

1 $$MSD({t}_{lag})=\frac{{L}^{2}}{3}(1-\,{e}^{-\frac{12{D}_{0}{t}_{lag}}{{L}^{2}}})$$

Due to the limited positional accuracy of the localization procedure, even immobile objects have an apparent diffusion-like mobility that determines the minimal detectable square displacement. In our case, the positional accuracy was ~28 nm which leads to a minimal detectable square displacement of 4× (28 nm)^2^ = 0,003136 µm^2^. This minimal detectable square displacement is indicated in the plot (Fig. 2E) by a horizontal line near the x-axis. Data analysis and simulations were performed with custom written software in MATLAB R2014b. For 2D cultured cells two 4 × 4 µm ROIs, for 3D cultured cells one 4 × 4 ROI per cell were analysed. In Fig. 4, in Fig. 5 and in supplementary table 1 medians of distributions of samples from the analyses parameters are visualized. All presented box plots (Figs 5 and S3, S6–18) show as a central line the median, the top and bottom of each box are the first and third quartile, top and bottom line represent the maximum and minimum values. Outliers are colored in red. Statistical analysis was performed with GraphPad Prism 7.

### Ripley’s K function analysis

For the analysis of the obtained single molecule data we used Ripley’s K function. This function identifies the average number of signals within concentric rings centered on each molecule^37, 38^.

2 $${\boldsymbol{K}}({\boldsymbol{r}}){\boldsymbol{=}}\frac{{\bf{1}}}{{\boldsymbol{n}}}\sum _{{\boldsymbol{i}}{\boldsymbol{=}}1}^{{\boldsymbol{n}}}{{\boldsymbol{N}}}_{{\boldsymbol{Pi}}}({\boldsymbol{r}}){\boldsymbol{/}}{\boldsymbol{\lambda }}$$

Where N is the number of points within the radius r of another point normalized by the number of points per area λ, where pi is the ith point summed over n points^39^. This function was linearized to obtain the so-called L-function and it was further normalized to generate the H-function.

3 $$L(r)=\sqrt{K(r)/\pi }$$

4 $$H(r)=L(r)-r$$

H(r) is 0 if the obtained signals are randomly, poisson distributed. A positive H(r) value indicates clustered data, a negative value indicates dispersed signals. To obtain the degree of clustering and the mean cluster radius the H-function was plotted against the length scale r. The first local maxima provides the information of the clustering (H(r)max) as well as the maximal cluster radius (rmax)^39^. For statistical analysis confidence intervals of 68.27% were generated by simulating 100 random distributions with the same number of signals as a control data set.

To visualize the clustering a 2-dimensional pseudo colored heat map was prepared similar to Williamson *et al*.^55^. Local L(r) values for each point were determined (with r = 130 nm) and interpolated as a surface plot by using the MATLAB interpolation function ‘v4’ with a grid set to 10 nm.

The total number of integrins were determined by counting all signals, molecules that convert to the same position were removed within the distance of the uncertainty of each dataset.

Cluster area, number of clusters per µm^2^, number of signals per cluster, cluster density (signals per area) and the ratio of clustered/unclustered signals were determined after generating binary cluster maps based on the publication of Owen *et al*.^56^. To generate binary cluster maps the threshold was adjusted for the controls and was set constant.

### Statistical analysis

Effects of ionizing radiation or ROS treatment on ECM-binding integrins and pFAK were analyzed for significance using an one-way ANOVA test with a Tukey post hoc test. Homogeneity of variances was tested with the Brown-Forsythe test, if this test resulted in a small P value the Kruskal-Wallis test with a Dunn’s multiple comparison test was used to determine significances. For both cases, p ≤ 0.05 was considered significant (*), p ≤ 0.01 very significant (**) and p ≤ 0.001 extremely significant (***). Also p ≤ 0.0001 (****) was noted.

## Electronic supplementary material

Supplementary information 
