# Supplementary material for: Direct evidence for cell adhesion-mediated radioresistance (CAM-RR) on the level of individual integrin β1 clusters
Source: Sci Rep. 2017 Jun 13;7:3393. doi: 10.1038/s41598-017-03414-4 (PMC5469790; doi:10.1038/s41598-017-03414-4)
Supplement: Supplementary file 1 — Supplementary information [file 41598_2017_3414_MOESM1_ESM.pdf]

Supplementary of:

Direct evidence for cell adhesion-mediated radioresistance (CAM-RR) on the level of individual integrin β1 clusters

Laura Babel[1,2], Miriam Grunewald[1,2], Robert Lehn[1], Markus Langhans[1], Tobias Meckel[1,2]

1 Membrane Dynamics, Department of Biology, Technische Universität Darmstadt, Germany
2 GRK 1657, Molecular and cellular responses to ionizing radiation, Technische Universität Darmstadt, Germany

β3 and αv integrins are affected by IR in an entirely different manner as β1 integrins

Results

Integrin heterodimers of αvβ1 and αvβ3 are well characterized ECM-binding integrins. To investigate if IR has an effect on integrins containing αv and β3 subunits comparative experiments were performed and results are displayed in in **S12 - 18** and in **S-table 1**. Both subunits showed a different reaction towards IR if compared to β1 integrins.

The most outstanding difference is shown in the effect of IR on integrins containing a β3 subunit. While the clustering (H max) shows a similar trend if compared to the results obtained from the β1 experiments, the severity of the initial effect 2 min after an irradiation with 2 Gy and 15 Gy of 2D cells is not comparable. R max is not affected if irradiated with 2 Gy and shows only a slight decrease after an irradiation with 15 Gy. An irradiation with 2 Gy of 3D cultured cells led to an increase in H max and r max, an irradiation with 15 Gy led only to a slight decrease in H max and had no effect on r max. In almost all parameters analyzed in **S-table 1** no or only slight differences were detected after an irradiation with 2 and 15 Gy of cells cultured in 2D or 3D. Only the number of molecules and the number of clusters increased significantly 2 min after an irradiation with 15 Gy of 2D cells.

Integrins containing a αv subunit of 2D cells show in their clustering (H max) and their cluster radius (r max) a comparable trend towards IR if compared to the β1 results seen in **Figure 4**. An irradiation with 2 Gy of 3D cultured cells led to an increase in H max with no significant effect on r max. 3D cells irradiated with 15 Gy showed no significant effect in H max as well as in r max (**S7**). The reaction of the parameters seen in **S-table 1** towards IR is comparable if the cells were cultured in 2D and irradiated with 2 and 15 Gy, only the number of clusters increases with the higher dose. All other parameters decreased and regenerated with time. The effect of 2 Gy on 3D cultured cells is comparable, but not as distinct as observed by 2D cultured cells. Surprisingly, the effects of IR on αv integrins of 3D cultured cells differ greatly if the cells was irradiated with 2 or 15 Gy. While an irradiation with 2 Gy led to a slight decrease in all parameters, an irradiation with 15 Gy led to an increase, while the ratio of clustered / unclustered signals of 3D cultured cells does not change. These results show that not only the ECM binding β1 integrins are affected by IR in dependence of the cell culture system, also integrins containing the αv or β3 subunit may contribute to CAM-RR.

Discussion

β3 integrins of 2D cultured cells are only slightly affected by an irradiation with 2 Gy (we could only detect a slight, still significant decrease in H max), an irradiation with 15 Gy had only a slight effect on the cluster radius but we could detect a significant decrease of the clustering 2 min after irradiation (**see S 12**). The most significant changes were detected in the number of molecule and clusters, both parameters increased 2 min after irradiation. Since the ratio of clustered / unclustered signals was constant as well as the cluster density and the signals per cluster, our results indicate that β3 integrins are upregulated and new clusters were generated. The effects of IR on β3 integrin differ in dependence of the cell culture system. 3D cultured cells irradiated with 2 Gy showed an increase in their clustering and cluster radius, but we could not detect distinct effects on the parameters in **S-table 1**, therefore we assume that β3 integrin clusters are compressed. Surprisingly, an irradiation with 15 Gy of 3D cultured cells revealed no such effects. Only a minor decrease of the clustering was detected. β3 integrin clusters of 3D cultured cells are affected after an irradiation with a low dose (2 Gy) but not after an irradiation with a high dose (15 Gy), 2D cultured cells are only affected after an irradiation with a higher dose (15 Gy) but show a completely different membrane located radiation response mechanismen.

Integrins containing the αv subunit are affected by IR in dependence of the dose and cell culture system. αv integrins of 2D cultured cells irradiated with 2 Gy showed a decrease in their clustering and cluster radius, as well as in all other parameter (except the number of clusters) from **S-table 1**. After an irradiation with 2 Gy we could detect more unclustered signals. Since the number of molecules decreased, we assume that the αv integrins emerge from the cluster by a combined axial and lateral membrane transport as reported for the β1 response mechanismen for higher doses. This mechanism also applies for 2D cells irradiated with 15 Gy. But, here we could also detect an increase in the number of clusters. This indicates, that the αv integrins do not simply emerge from the the clusters, they also form new clusters - αv clusters fragment after irradiation with 15 Gy of 2D cultured cells. An irradiation with 2 Gy on 3D cultured cells had the same effect on αv and β3 integrins: the clustering and the cluster radius increase, leading to a compression of the clusters by a lateral diffusion. The effects of 15 Gy on αv and β3 integrins of 3D cultured cells is not comparable: only minor decreases in the clustering and the cluster radius of αv integrins were detected, the number of molecules and clusters as well as the signals per cluster increased after IR. αv integrins are upregulated and form new clusters after irradiation.

Supplementary figures

S-table1: Percental change of various parameter after IR of β3 and αv integrins of 2D and 3D cultured MEF cells.

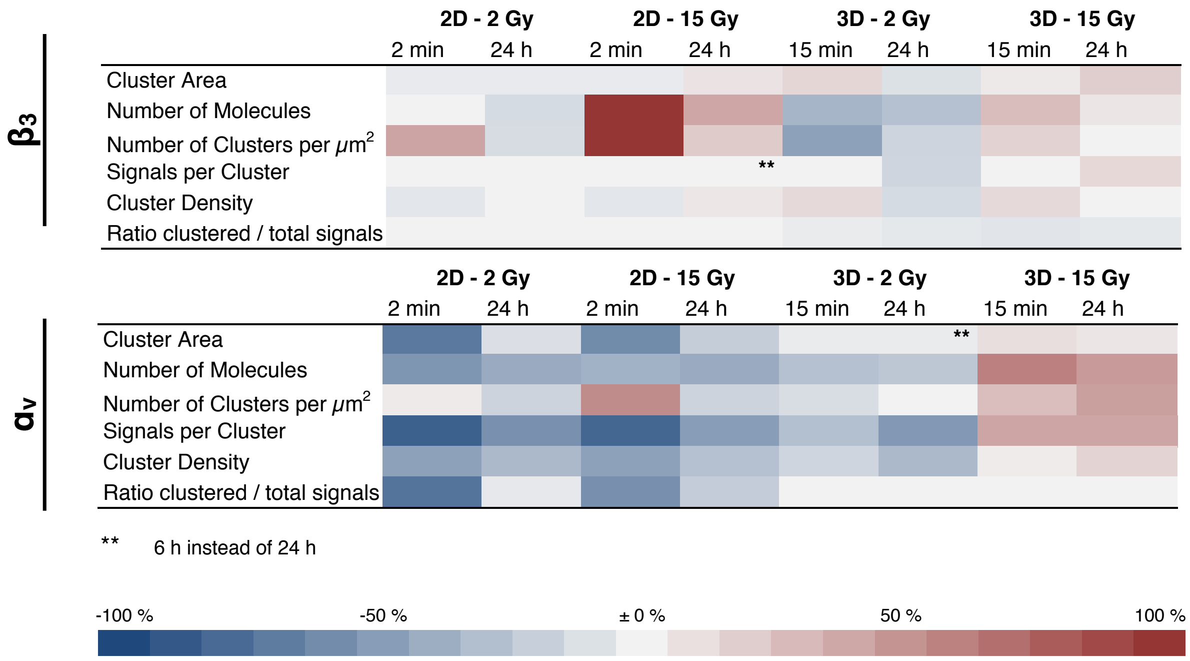

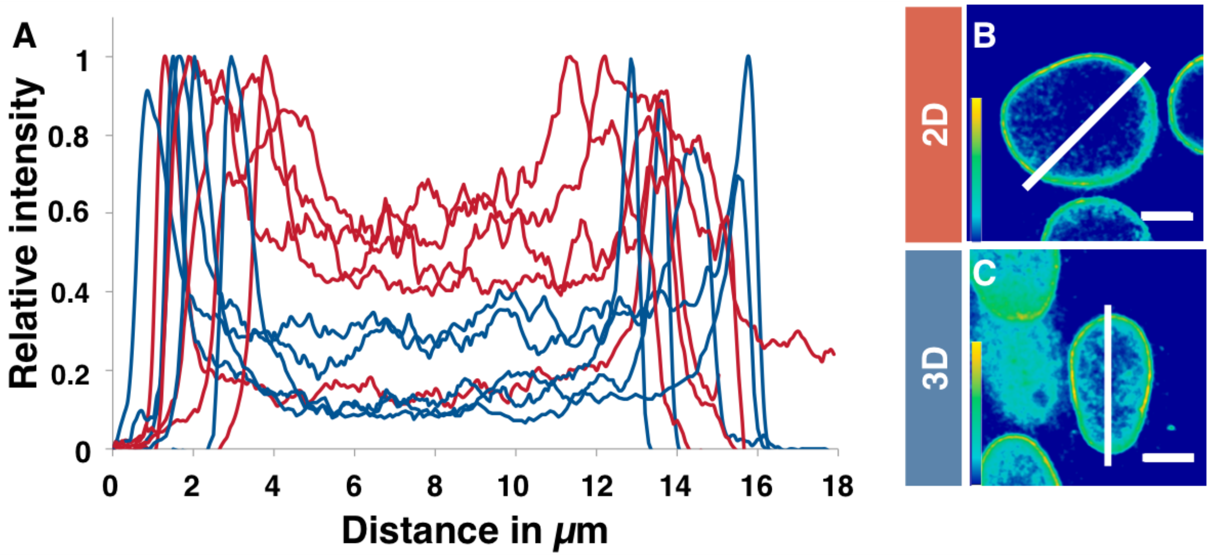

S 1: Line profiles of Lamin A/C of 2D (red) and 3D (blue) cultured cells. (A) Relative intensities of line profiles with a width of 3.5 µm of a Lamin A/C staining from cells cultured in 2D (red) and 3D (blue). N=1, n=5 for 2D and 3D cultured cells. (B, C) Heat map of a Lamin A/C immunostaining of 2D (B) and 3D (C) cultured cells including an exemplary line profile plotted in (A). Scale bar is 5 µm.

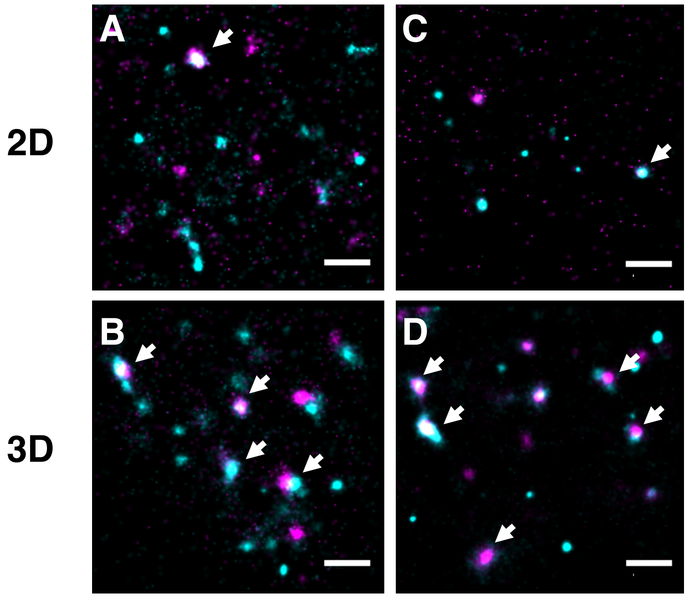

S 2: Colocalization of integrins of MEF cells cultured in 2D or 3D. (A) α_v (magenta) and β_1 (cyan) of 2D cultured cells. (B) Corresponding data for 3D cultured cells. (C) α_v (magenta) and β_3 (cyan) of 2D cultured cells. (D) Corresponding data for 3D cultured cells. Scale bar is 2 µm. Arrows indicate integrin-subunit colocalization (white).

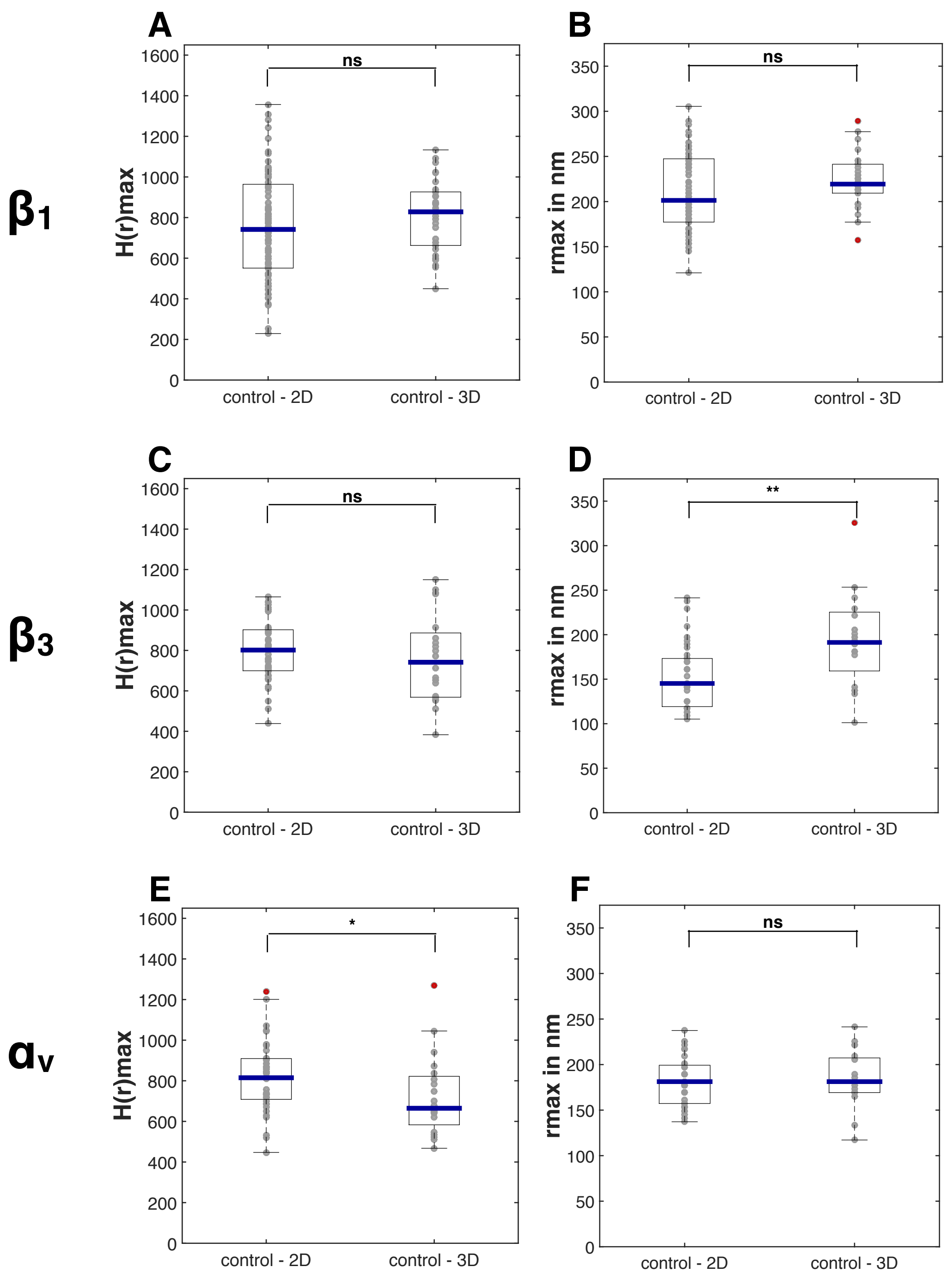

S 3: Clustering and cluster radius of all controls of β_1, β_3 and α_V integrins. (A) Box plot of the clustering (H(r)max) of β_1 integrins of 2D (N=8, n=80) and 3D (N=6, n=30) cultured control cells. (B) Box plot of the cluster radius r of β_1 integrins of 2D (N=8, n=80) and 3D (N=6, n=30) cultured control cells. (A, B) Statistical analysis was performed with an ordinary one-way ANOVA. (C) Box plot of the clustering (H(r)max) of β_3 integrins of 2D (N=4, n=40) and 3D (N=4, n=20) cultured control cells. (D) Box plot of the cluster radius r of β_3 integrins of 2D (N=4, n=40) and 3D (N=4, n=10) cultured control cells. (C, D) Statistical analysis was performed with an ordinary one-way ANOVA. (E) Box plot of the clustering (H(r)max) of α_v integrins of 2D (N=4, n=40) and 3D (N=4, n=20) cultured control cells. (F) Box plot of the cluster radius r of α_V integrins of 2D (N=4, n=40) and 3D (N=4, n=20) cultured control cells. (E, F) Statistical analysis was performed with a Kruskal - Wallis test. *p ≤ 0.05, **p ≤ 0.01. NS, not significantly different.

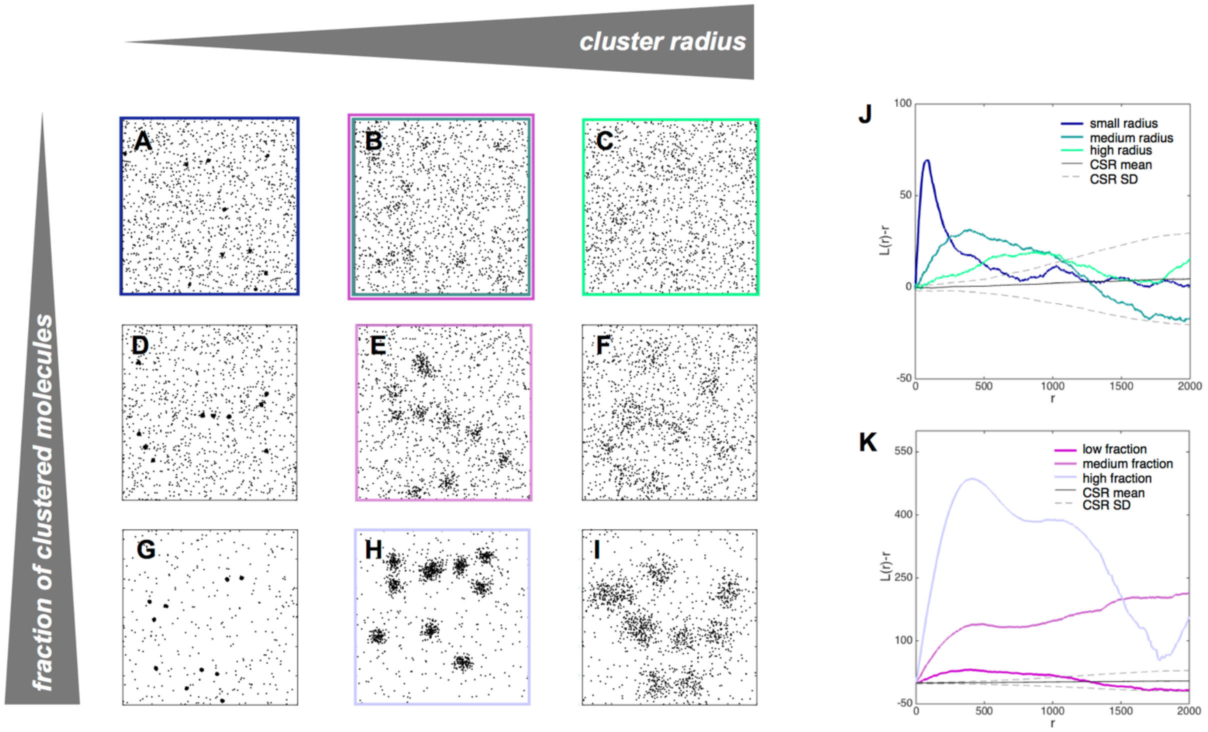

S 4: Simulated data demonstrating the influence of cluster radius and the fraction of clustered molecules on the H-function. While the cluster radius is depicted as the first maximum of the H-function, the clustering degree correlates with the value of L(r)-r. (A-I) Simulated distributions of 1500 molecules including 10 clusters with varying cluster radii and clustering degrees. In the upper row (A-C) the 10 cluster each consist of 20 molecules, in the middle row (D-E) clusters contain 50 molecules and in the lower row (G-I) clusters comprise 120 molecules. The cluster radius increases from left to right with a small radius in the left column (A, D and G), a medium radius for the middle column (B, E and H) and large radius in the right column (C, F and I). J and K show the corresponding H functions for the upper row (J) and the middle column (K) and the mean and standard deviation of 1500 completely random distributed molecules from 100 simulations. The cluster radius is depicted in the H-plot as the first maximum of the H-function at the scale of the highest clustering degree. Hence for expanding cluster radii r max increases (J). With higher cluster ratios the maximum of L(r)-r increases (K). The radius of clusters against a background of unclustered molecules likewise impacts the clustering degree, hence in J L(r)-r max decreases with higher cluster radii.

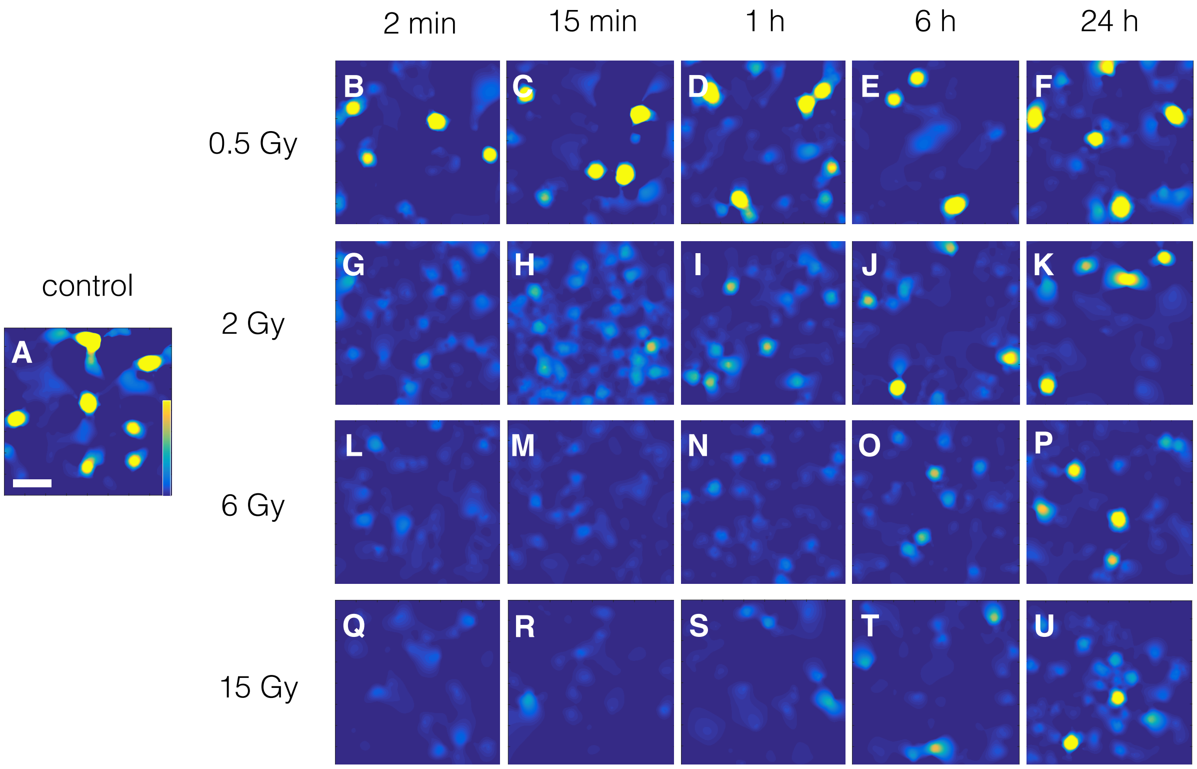

S 5: Effects of IR on the clustering of β_1 integrins of 2D cultured cells. Heat maps visualising unclustered (dark blue) and clustered (yellow) regions. Cells were irradiated with 0.5, 2, 6 and 15 Gy and were fixed 2 min, 15 min, 1 h, 6 h and 24 h after irradiation. (A) Control. (B-F) Cells irradiated with 0.5 Gy. (G-K) Cells irradiated with 2 Gy. (L-P) Cells irradiated with 6 Gy and (Q-U) cells irradiated with 15 Gy. Scale bar is 1 µm.

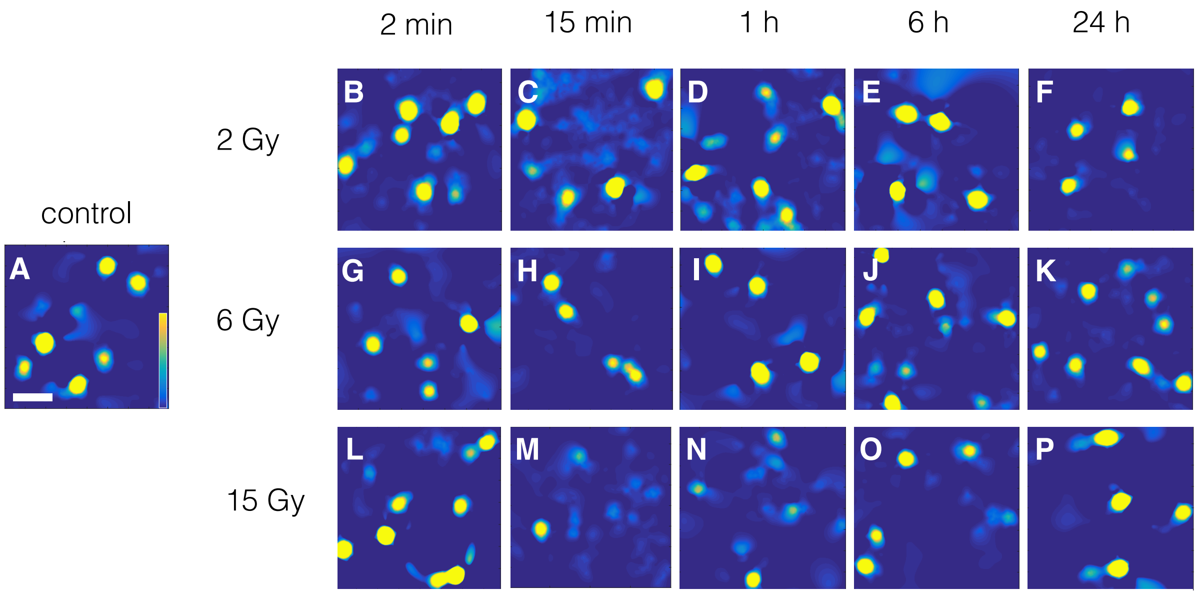

S 6: Effects of IR on the clustering of β_1 integrins of 3D cultured cells. Heat maps visualising unclustered (dark blue) and clustered (yellow) regions. Cells were irradiated with 2, 6 and 15 Gy and were fixed 2 min, 15 min, 1 h, 6 h and 24 h after irradiation. (A) Control. (B-D) Cells irradiated with 2 Gy. (G-K) Cells irradiated with 6Gy. (L-P) Cells irradiated with 15 Gy. Scale bar is 1 µm.

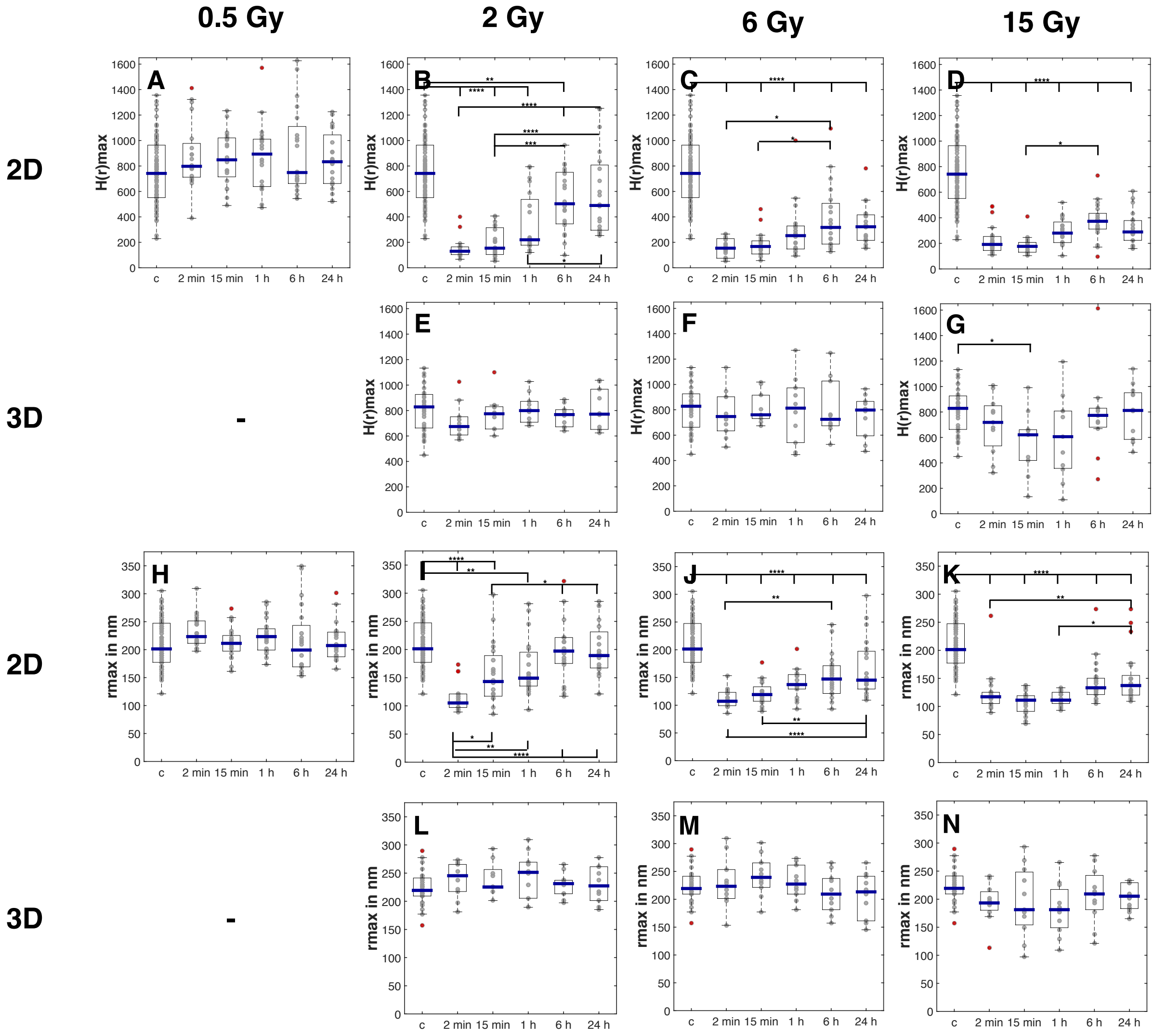

S 7: Effects of IR on the clustering (A-G) and the cluster radius (H-N) of β_1 integrins of 2D and 3D cultured cells. Box plots of the clustering H(r)max or the cluster radius r in nm plotted against the time (control (c), 2 min, 15 min, 1 h, 6 h and 24 h). 2D cultured cells were irradiated with 0.5, 2, 6 and 15 Gy (A-D, H-K), 3D cultured cells were irradiated with 2, 6 and 15 Gy (E-G, L-N). For 2D cells N=2, n=20. controls were pooled, N=8, n=80. For 3D cells N=2, n=10. Controls were pooled, N=6, n=30. Statistical analysis was performed with an ordinary one-way ANOVA (A, D, E, F, G, H, I, K, L, M) or with a Kruskal -Wallis test (B, C, F, N). *p ≤ 0.05, **p ≤ 0.01,***p ≤ 0.001 and ****p ≤ 0.0001. If not further noted no significance was detected.

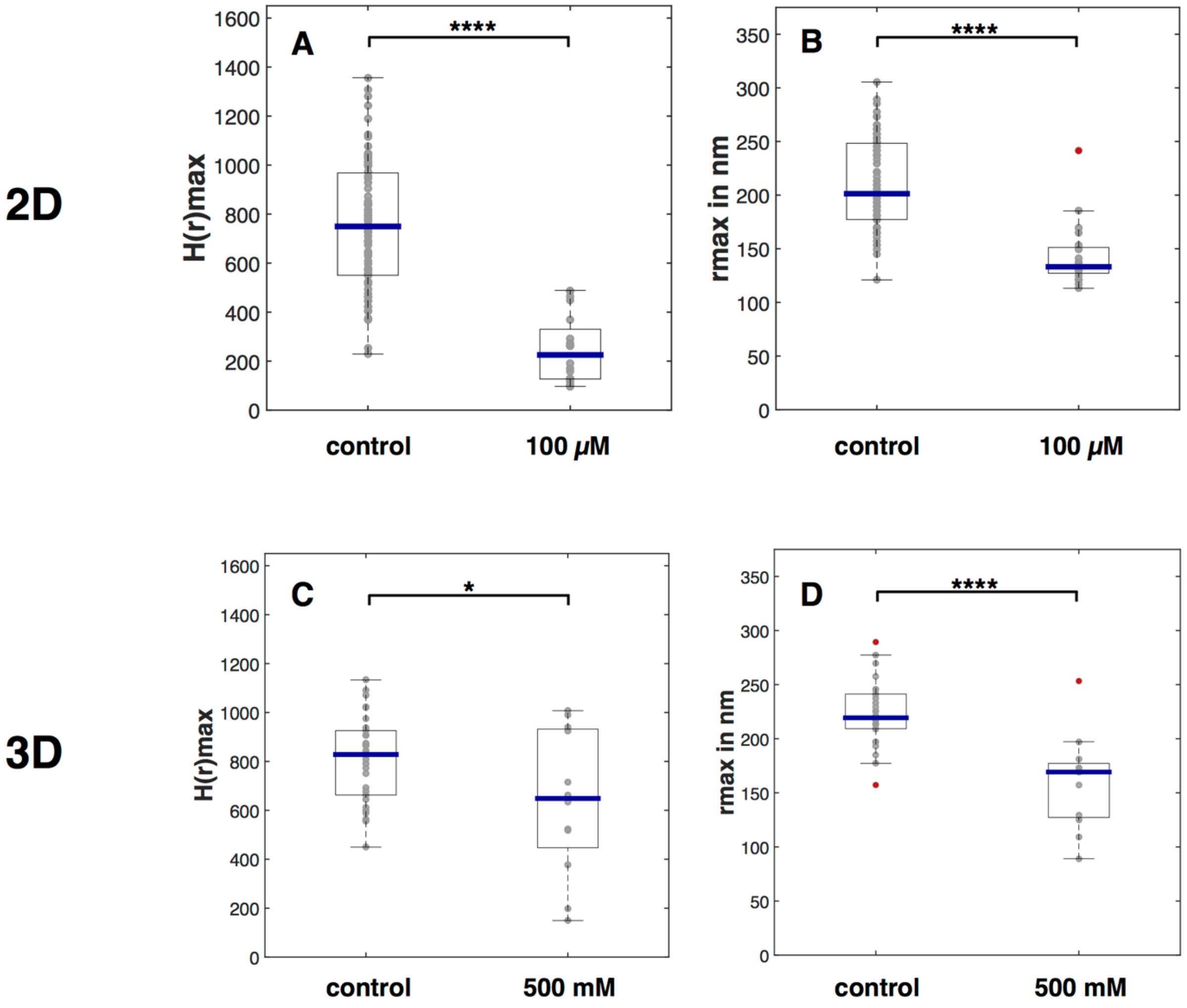

S 8: Cluster disintegration is induced by ROS. Box plots of the clustering H(r)max (A, C) and the cluster radius r in nm (B, D) of controls and cells treated with 100 µM H_2O_2 (2D, A and B) or of controls and cells treated with 500 mM H_2O_2 (3D, C and D). N=1, n=20. Statistical analysis was performed with a two-tailed Mann-Whitney test for (A) or with a two-tailed, unpaired t-test for (B, C, D). $^*p \leq 0.05$, $^{****}p \leq 0.0001$.

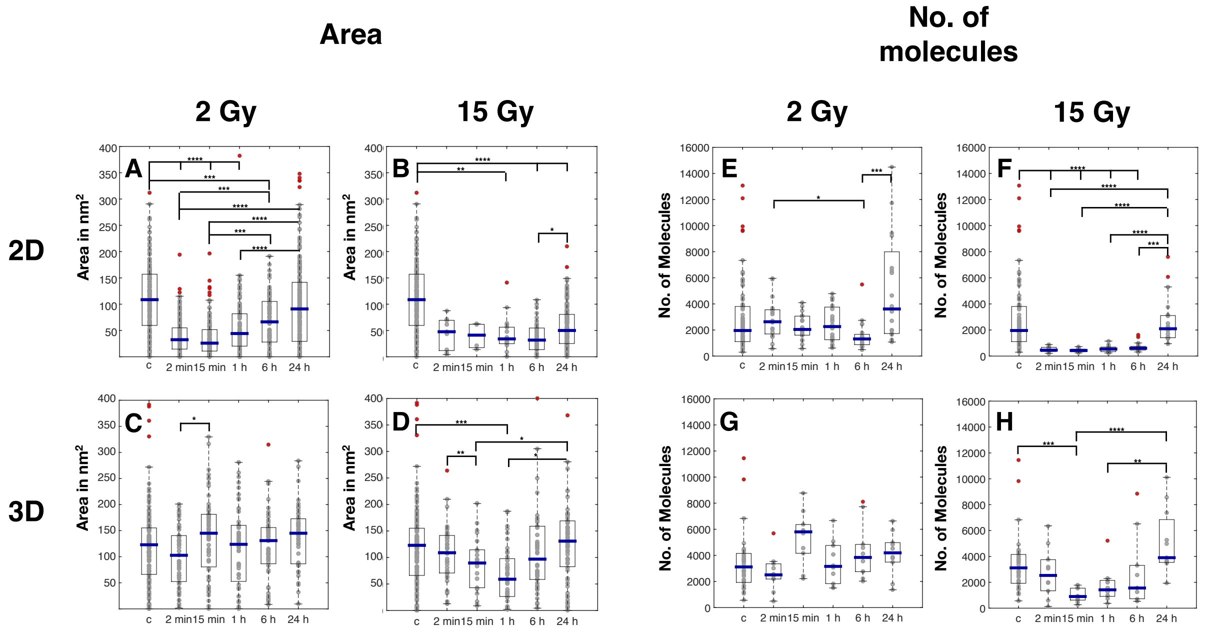

S 9: Effects of IR on the cluster area (A-D) and the number of molecules per ROI (E-H) of β_1 integrins of 2D (A/B, E/F) and 3D (C/D, G/H) cultured cells. Box plots of the cluster area and the number of molecules, plotted against the time (2 min, 15 min, 1 h, 6 h, 24 h and the control (c)). Cells were irradiated with 2 or 15 Gy. For 2D cells N=2, n=20. controls were pooled, N=8, n=80. For 3D cells N=2, n=10. Controls were pooled, N=6, n=30. Statistical analysis was performed with an ordinary one-way ANOVA (B, C, D, H) or with a Kruskal - Wallis test (A, E, F, G). $*p \leq 0.05$, $**p \leq 0.01$, $***p \leq 0.001$ and $****p \leq 0.0001$. If not further noted no significance was detected.

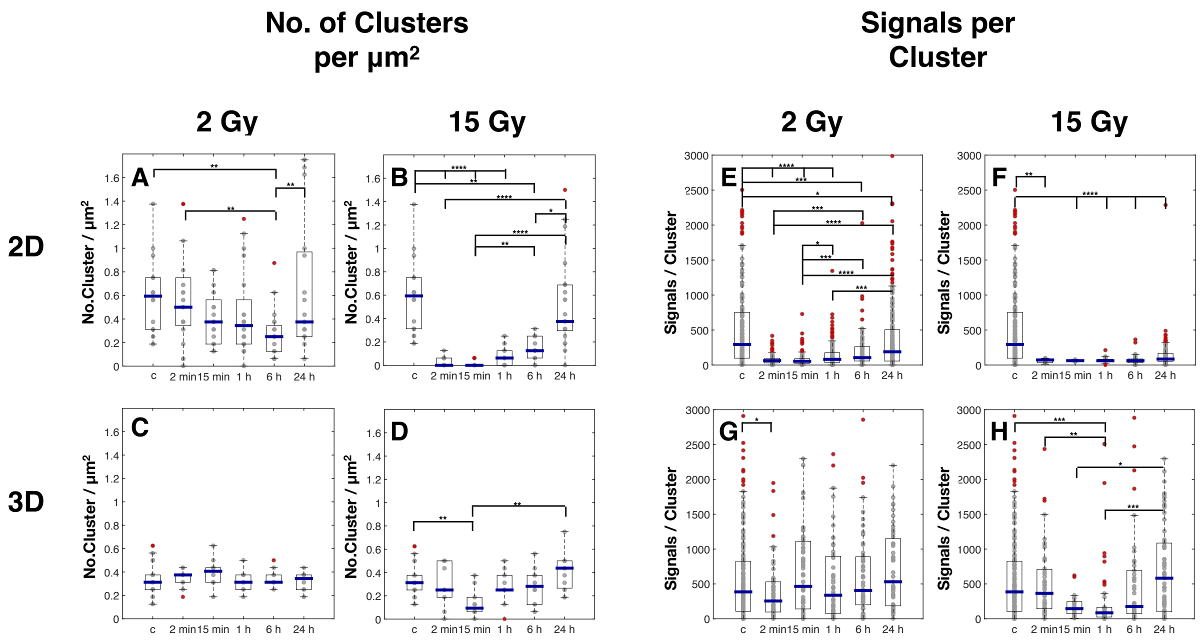

S 10: Effects of IR on the number of clusters per μm^2 (A-D) and the number of signals per cluster (E-H) of β_1 integrins of 2D (A/B, E/F) and 3D (C/D, G/H) cultured cells. Box plots of the number of clusters per μm^2 and the number of signals per cluster, plotted against the time (2 min, 15 min, 1 h, 6 h, 24 h and the control (c)). Cells were irradiated with 2 or 15 Gy. For 2D cells N=2, n=20. controls were pooled, N=8, n=80. For 3D cells N=2, n=10. Controls were pooled, N=6, n=30. Statistical analysis was performed with an ordinary one-way ANOVA (A, C, D, E, F, H) or with a Kruskal - Wallis test (B, G). $*p \leq 0.05$, $**p \leq 0.01$, $***p \leq 0.001$ and $****p \leq 0.0001$. If not further noted no significance was detected.

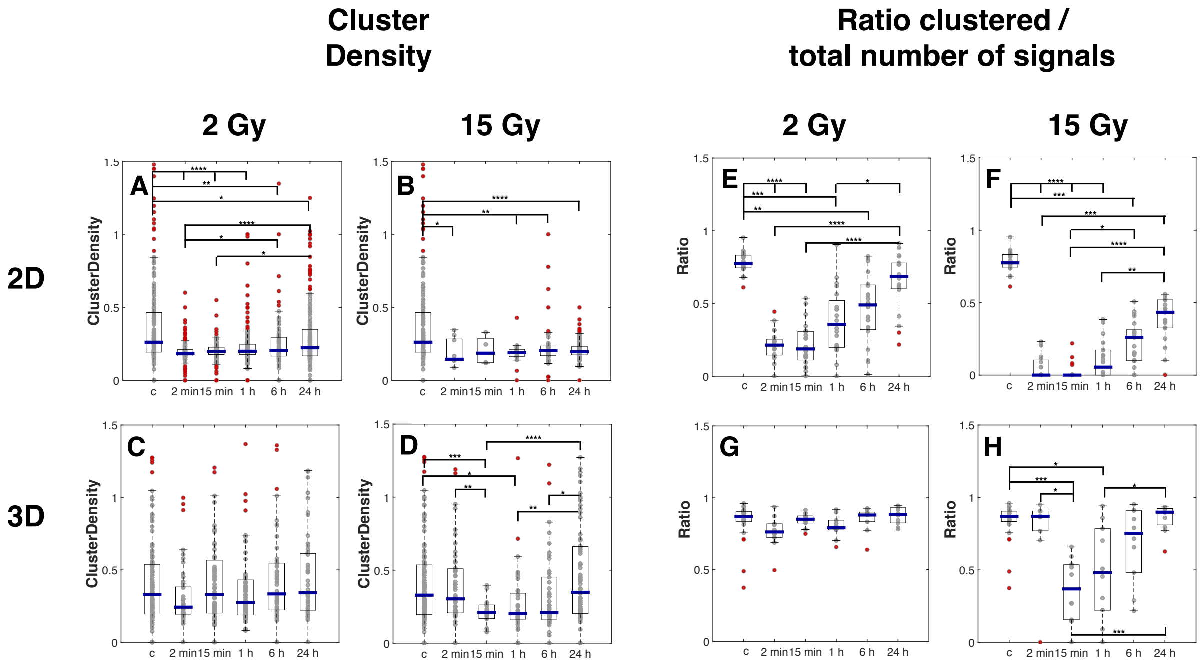

S 11: Effects of IR on the cluster density (A-D) and the ratio of clustered / total number of signals (E-H) of β_1 integrins of 2D (A/B, E/F) and 3D (C/D, G/H) cultured cells. Box plots of the cluster density and the ratio of clustered/unclustered signals, plotted against the time (2 min, 15 min, 1 h, 6 h, 24 h and the control (c)). Cells were irradiated with 2 or 15 Gy. For 2D cells N=2, n=20. controls were pooled, N=8, n=80. For 3D cells N=2, n=10. Controls were pooled, N=6, n=30. Statistical analysis was performed with an ordinary one-way ANOVA (C, G) or with a Kruskal - Wallis test (A, B, E, F, H). *$p \leq 0.05$, **$p \leq 0.01$,***$p \leq 0.001$ and ****$p \leq 0.0001$. If not further noted no significance was detected.

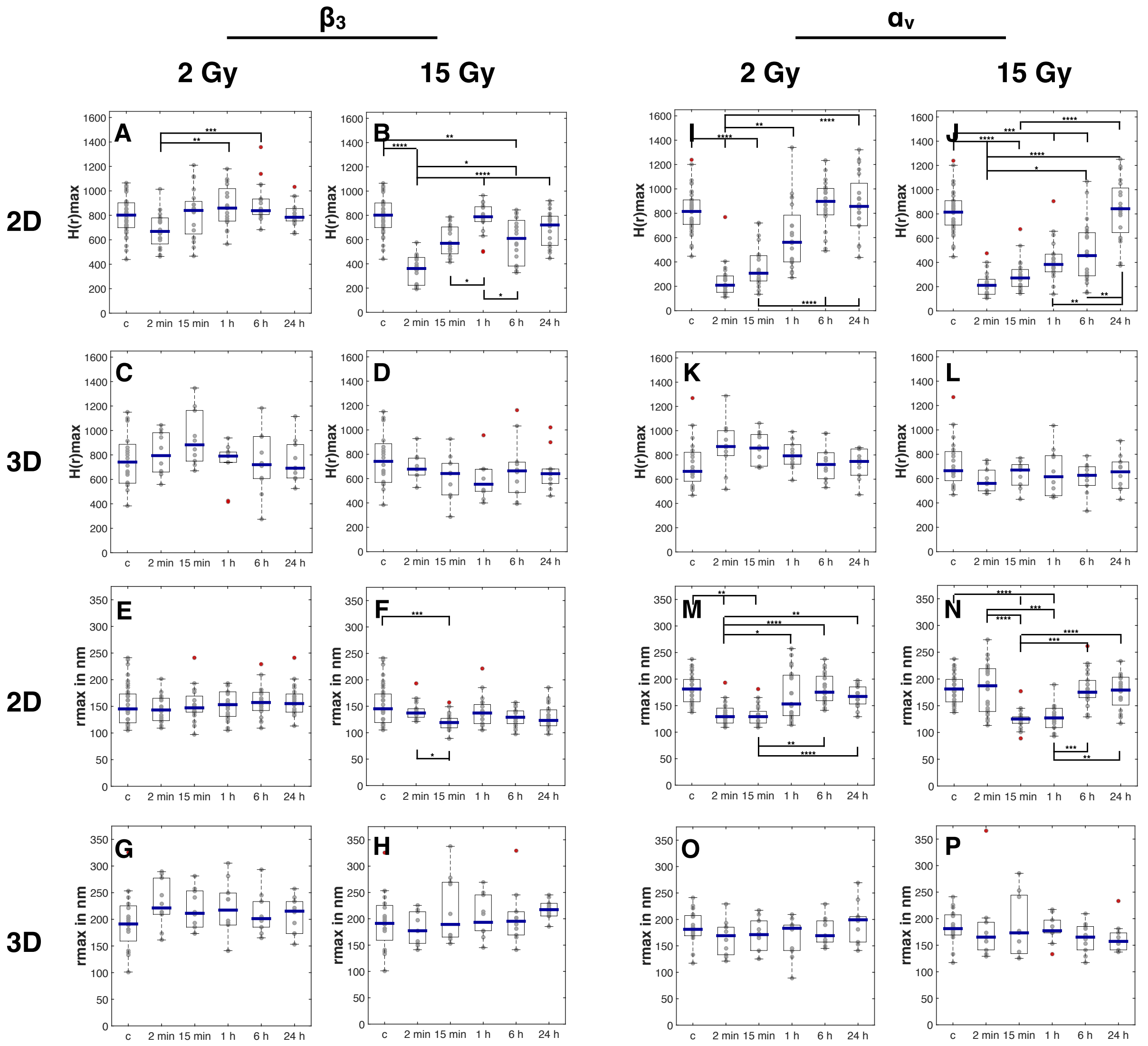

S 12: Effects of IR on the clustering and the cluster radius of β_3 (A-H) and α_V (I-P) integrins of 2D (A/B, e/F, I/J, O/P) and 3D (C/D, E/F, K/L, M/N) cultured cells. Box plot of the clustering H(r) max or the cluster radius r in nm plotted against the time (control (c), 2 min, 15 min, 1 h, 6 h and 24 h). Cells were irradiated with 2 or 15 Gy. For 2D cells N=2, n=20. controls were pooled, N=4, n=40. For 3D cells N=2, n=10. Controls were pooled, N=4, n=20. Statistical analysis was performed with an ordinary one-way ANOVA (A, C, D, E, G, H, K, L, O, P) or with a Kruskal - Wallis test (B, F, I, J, M, N). *$p \leq 0.05$, **$p \leq 0.01$,***$p \leq 0.001$ and ****$p \leq 0.0001$. If not further noted no significance was detected.

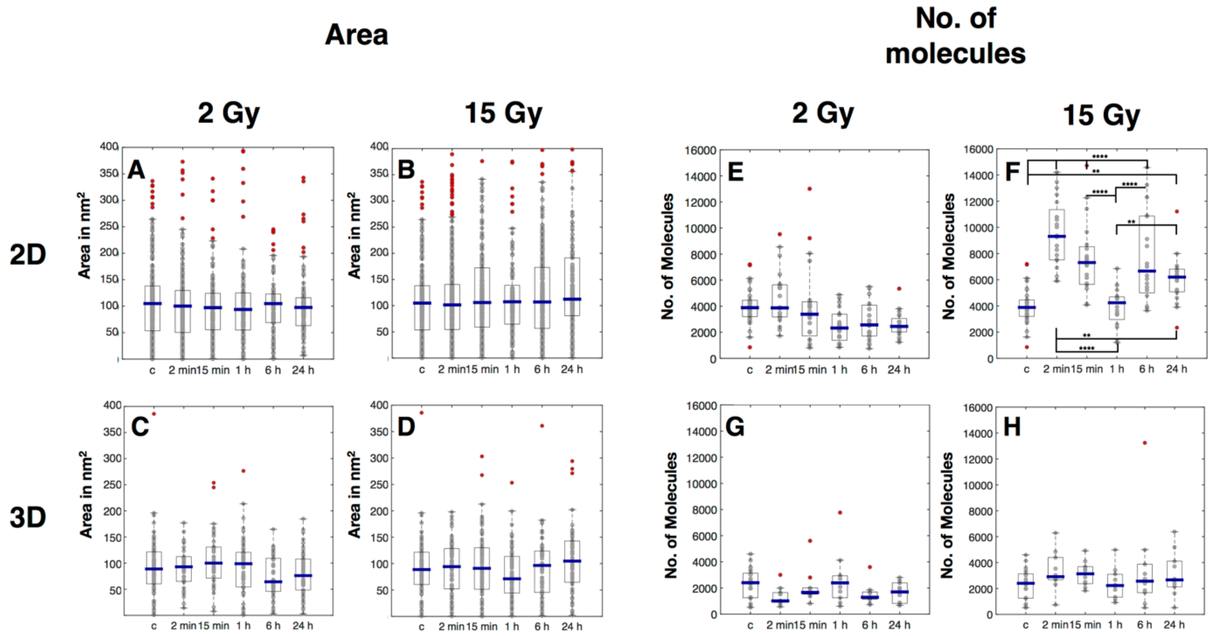

S 13: Effects of IR on the cluster area (A-D) and the number of molecules per ROI (E-H) of β_3 integrins of 2D (A/B, E/F) and 3D (C/D, G/H) cultured cells. Box plots of the cluster area and the number of molecules, plotted against the time (2 min, 15 min, 1 h, 6 h, 24 h and the control (c)). Cells were irradiated with 2 or 15 Gy. For 2D cells N=2, n=20. controls were pooled, N=4, n=40. For 3D cells N=2, n=10. Controls were pooled, N=4, n=20. Statistical analysis was performed with an ordinary one-way ANOVA (A - H). *p ≤ 0.05, **p ≤ 0.01,***p ≤ 0.001 and ****p ≤ 0.0001. If not further noted no significance was detected.

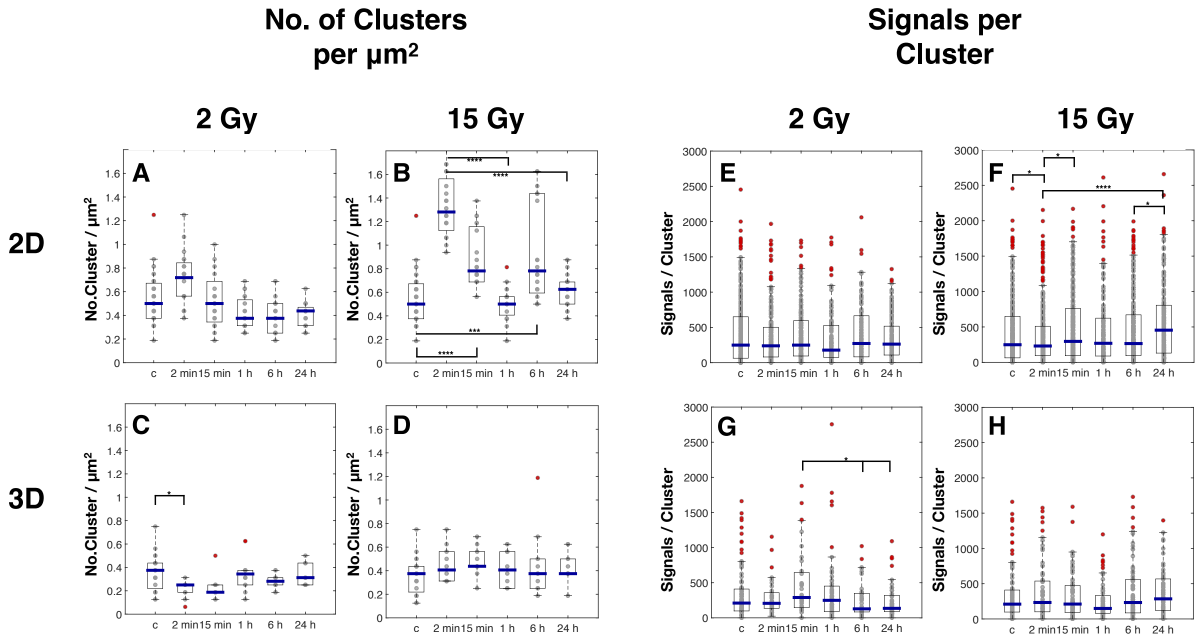

S 14: Effects of IR on the number of clusters per µm^2 (A-D) and the number of signals per cluster (E-H) of β_3 integrins of 2D (A/B, E/F) and 3D (C/D, G/H) cultured cells. Box plots of the number of clusters per µm^2 and the number of signals per cluster, plotted against the time (2 min, 15 min, 1 h, 6 h, 24 h and the control (c)). Cells were irradiated with 2 or 15 Gy. For 2D cells N=2, n=20. controls were pooled, N=4, n=40. For 3D cells N=2, n=10. Controls were pooled, N=4, n=20. Statistical analysis was performed with an ordinary one-way ANOVA (A, C - H) or with a Kruskal - Wallis test (B). *p ≤ 0.05, **p ≤ 0.01,***p ≤ 0.001 and ****p ≤ 0.0001. If not further noted no significance was detected.

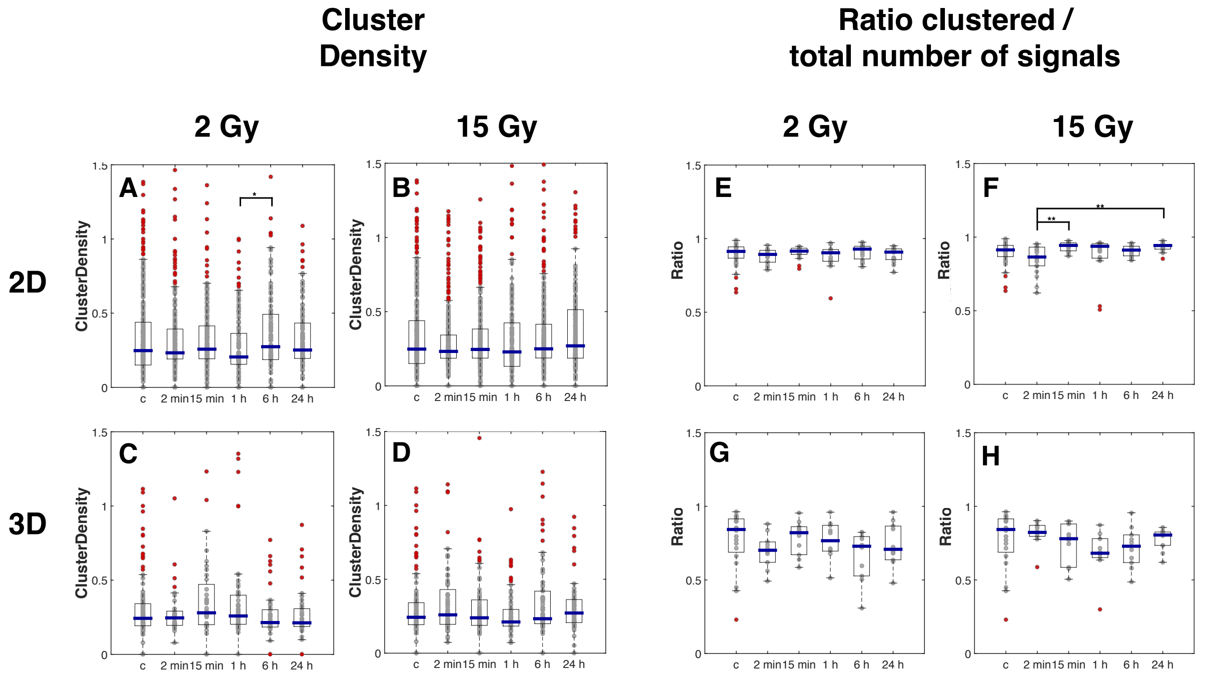

S 15: Effects of IR on the cluster density (A-D) and the ratio of clustered / total number of signals (E-H) of β_3 integrins of 2D (A/B, E/F) and 3D (C/D, G/H) cultured cells. Box plots of the cluster density and the ratio of clustered/unclustered signals, plotted against the time (2 min, 15 min, 1 h, 6 h, 24 h and the control (c)). Cells were irradiated with 2 or 15 Gy. For 2D cells N=2, n=20. controls were pooled, N=4, n=40. For 3D cells N=2, n=10. Controls were pooled, N=4, n=20. Statistical analysis was performed with an ordinary one-way ANOVA (A, C, D, E, G, H) or with a Kruskal - Wallis test (B, F). *$p \leq 0.05$, **$p \leq 0.01$,***$p \leq 0.001$ and ****$p \leq 0.0001$. If not further noted no significance was detected.

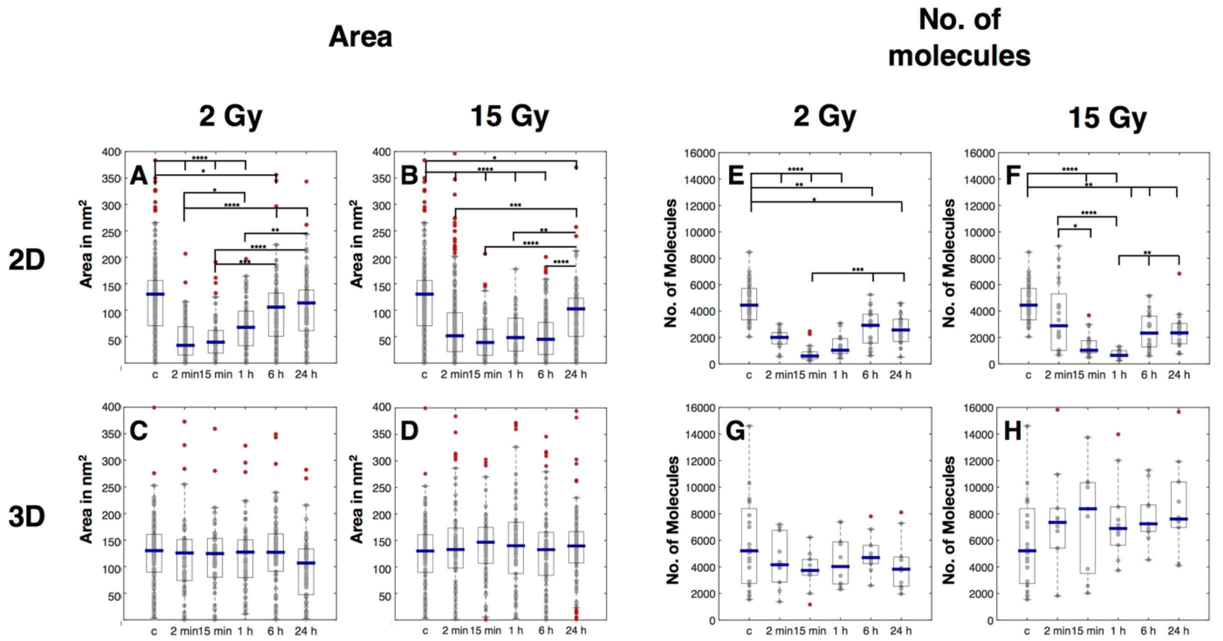

S 16: Effects of IR on the cluster area (A-D) and the number of molecules per ROI (E-H) of α_V integrins of 2D (A/B, E/F) and 3D (C/D, G/H) cultured cells. Box plots of the cluster area and the number of molecules, plotted against the time (2 min, 15 min, 1 h, 6 h, 24 h and the control (c)). Cells were irradiated with 2 or 15 Gy. For 2D cells N=2, n=20. controls were pooled, N=4, n=40. For 3D cells N=2, n=10. Controls were pooled, N=4, n=20. Statistical analysis was performed with an ordinary one-way ANOVA (C, G, H) or with a Kruskal - Wallis test (A, B, D, E, F). $*p \leq 0.05$, $**p \leq 0.01$,$***p \leq 0.001$ and $****p \leq 0.0001$. If not further noted no significance was detected.

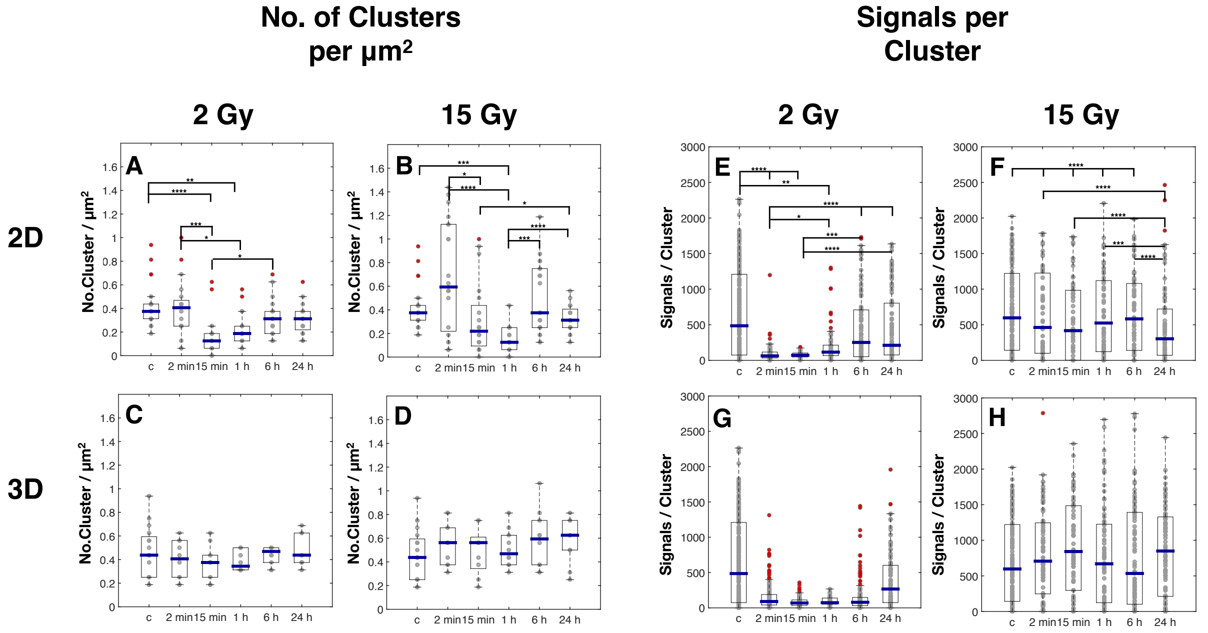

S 17: Effects of IR on the number of clusters per µm^2 (A-D) and the number of signals per cluster (E-H) of α_V integrins of 2D (A/B, E/F) and 3D (C/D, G/H) cultured cells. Box plots of the number of clusters per µm^2 and the number of signals per cluster, plotted against the time (2 min, 15 min, 1 h, 6 h, 24 h and the control (c)). Cells were irradiated with 2 or 15 Gy. For 2D cells N=2, n=20. controls were pooled, N=4, n=40. For 3D cells N=2, n=10. Controls were pooled, N=4, n=20. Statistical analysis was performed with an ordinary one-way ANOVA (A, C, D, G, H) or with a Kruskal - Wallis test (B, E, F). *p ≤ 0.05, **p ≤ 0.01,***p ≤ 0.001 and ****p ≤ 0.0001. If not further noted no significance was detected.

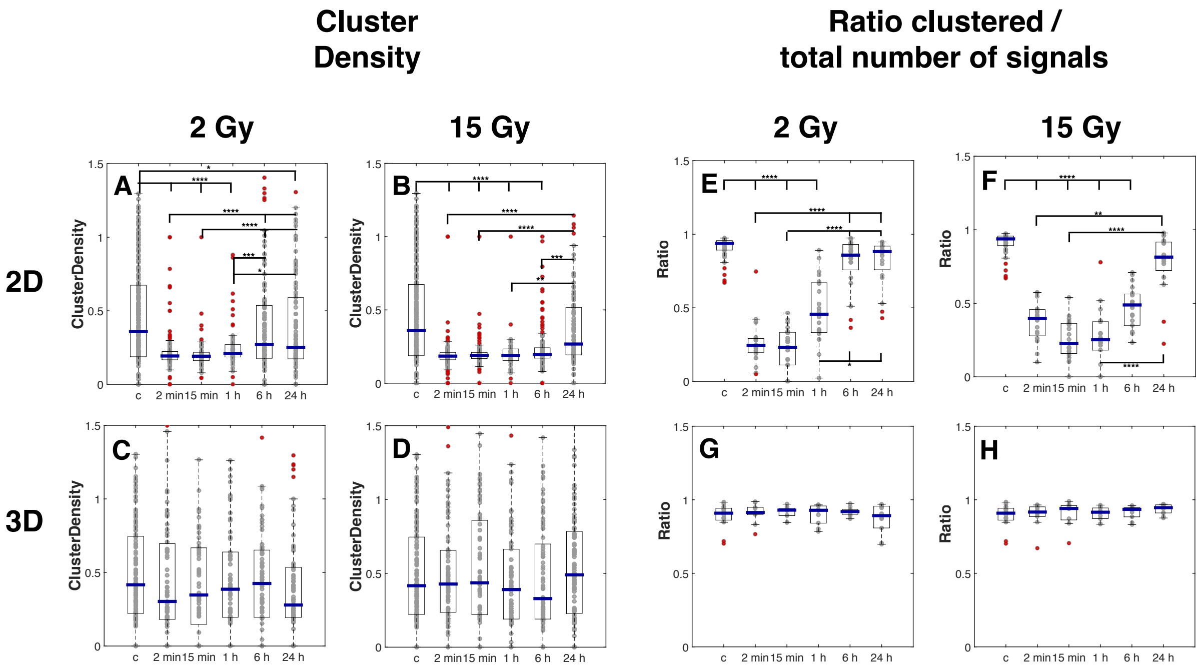

S 18: Effects of IR on the cluster density (A-D) and the ratio of clustered / total number of signals (E-H) of α_V integrins of 2D (A/B, E/F) and 3D (C/D, G/H) cultured cells. Box plots of the cluster density and the ratio of clustered/unclustered signals, plotted against the time (2 min, 15 min, 1 h, 6 h, 24 h and the control (c)). Cells were irradiated with 2 or 15 Gy. For 2D cells N=2, n=20. controls were pooled, N=4, n=40. For 3D cells N=2, n=10. Controls were pooled, N=4, n=20. Statistical analysis was performed with an ordinary one-way ANOVA (C, D, G, H) or with a Kruskal - Wallis test (A, B, E, F). *$p \leq 0.05$, **$p \leq 0.01$,***$p \leq 0.001$ and ****$p \leq 0.0001$. If not further noted no significance was detected.

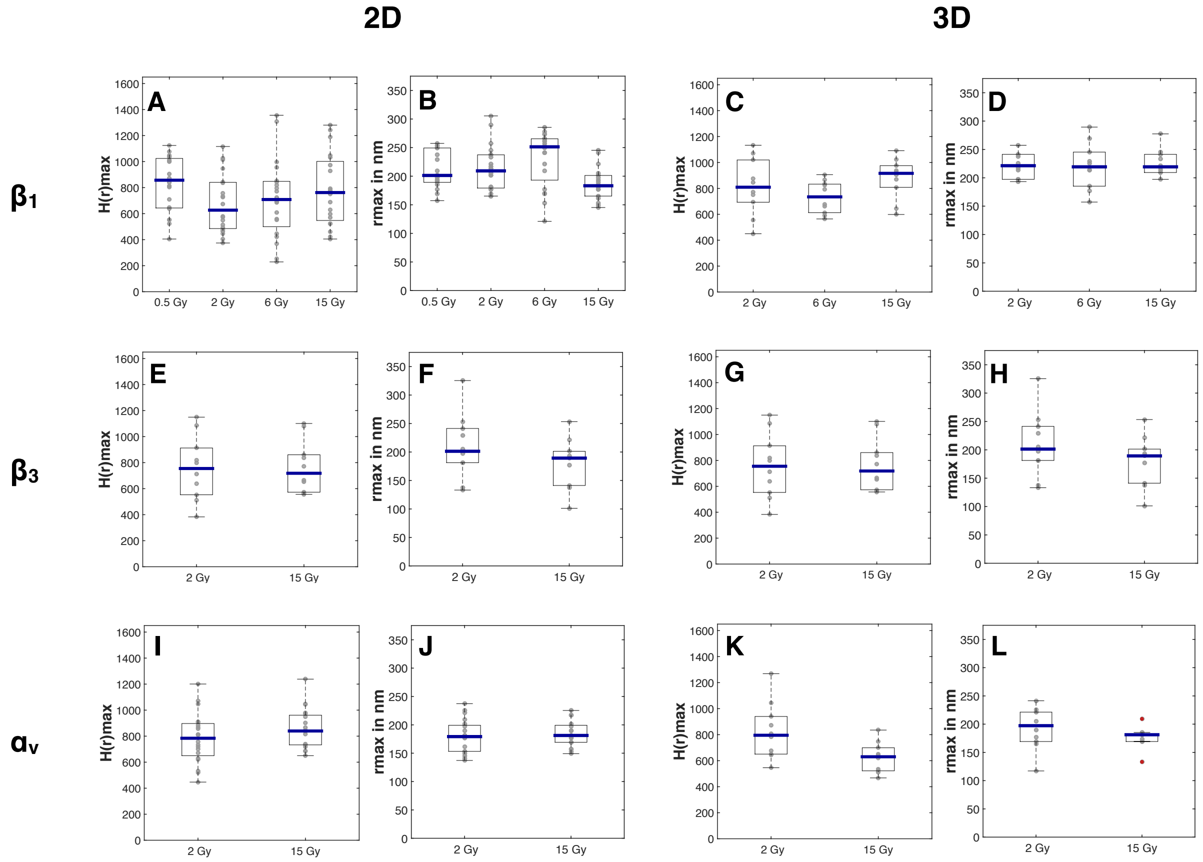

S 19: All pooled controls of β_1(A-D), β_3 (E-H) and α_V (I-L) integrins of 2D (A/B, E/F, I,J) and 3D cultured cells (C/D, G/H, K/L). Box plots of the clustering H(r)max and the cluster radius r in nm plotted against the dose. Cells stained for β_1 integrin were irradiated with 0.5, 2, 6 and 15 Gy if cultured in 2D, 3D cultured cells were irradiated with 2, 6 and 15 Gy. 2D and 3D cultured cells stained for β_3 and α_V were irradiated with 2 and 15 Gy. For 2D cells N=2, n=20. For 3D cells N=2, n=10.
